# KLRG1 identifies regulatory T cells with mitochondrial alterations that accumulate with aging

**DOI:** 10.1038/s43587-025-00855-9

**Published:** 2025-04-30

**Authors:** Gonzalo Soto-Heredero, Enrique Gabandé-Rodríguez, Elisa Carrasco, José Ignacio Escrig-Larena, Manuel M. Gómez de las Heras, Sandra Delgado-Pulido, Isaac Francos-Quijorna, Eva M. Blanco, Álvaro Fernández-Almeida, David Abia, María Josefa Rodríguez, Cristina M. Fernández-Díaz, María Beatriz Álvarez-Flores, Ana Ramírez de Molina, Sascha Jung, Antonio del Sol, Virginia Zorita, Fátima Sánchez-Cabo, Carlos Torroja, María Mittelbrunn

**Affiliations:** 1 Departamento de Biología Molecular, Facultad de Ciencias, Centro de Biología Molecular ‘Severo Ochoa’, Universidad Autónoma de Madrid, Madrid, Spain; 2 Consejo Superior de Investigaciones Científicas, Centro de Biología Molecular ‘Severo Ochoa’, Universidad Autónoma de Madrid, Madrid, Spain; 3 Departamento de Biología, Facultad de Ciencias, Centro de Biología Molecular ‘Severo Ochoa’, Universidad Autónoma de Madrid, Madrid, Spain; 4 Servicio de Microscopía Electrónica, Centro de Biología Molecular ‘Severo Ochoa’, Universidad Autónoma de Madrid, Madrid, Spain; 5 IMDEA Food Institute, CEI UAM+CSIC, Madrid, Spain; 6 Centro Nacional de Investigaciones Cardiovasculares, Instituto de Salud Carlos III, Madrid, Spain; 7 Luxembourg Centre for Systems Biomedicine, University of Luxembourg, Esch sur-Alzette, Luxembourg; 8 CIC bioGUNE-BRTA (Basque Research and Technology Alliance), Bizkaia Technology Park, Derio, Spain; 9 IKERBASQUE, Basque Foundation for Science, Bilbao, Spain

**Keywords:** Lymphocytes, Senescence, Metabolism, Ageing, Inflammation

## Abstract

Recent studies using single-cell RNA sequencing technology have uncovered several subpopulations of CD4^+^ T cells that accumulate with aging. These age-associated T cells are emerging as relevant players in the onset of inflammaging and tissue senescence. Here, based on information provided by single-cell RNA sequencing data, we present a flow cytometry panel that allows the identification of age-associated T cell subsets in systematic larger analysis in mice. We use this panel to evaluate at the single-cell level mitochondrial and senescence marks in the different age-associated CD4^+^ T cell subpopulations. Our analysis identifies a subpopulation of regulatory T (T_reg_) cells that is characterized by the extracellular expression of the co-inhibitory molecule killer cell lectin-like receptor subfamily G member 1 (KLRG1) and accumulates with aging in humans and mice. KLRG1-expressing T_reg_ cells display senescence features such as mitochondrial alterations, increased expression of cell-cycle regulators and genomic DNA damage. Functionally, KLRG1^+^ T_reg_ cells show a reduced suppressive activity in vivo accompanied by a pro-inflammatory phenotype.

## Abstract (web summary)

T cells undergo age-related changes that impair their organismal functions. Here Soto-Heredero et al. show that regulatory T cells characterized by the expression of KLRG1 accumulate with age in both mice and humans and exhibit features including mitochondrial decline and an inflammatory phenotype.

## Main

With age, the immune system loses the ability to respond to infections, cancer or vaccination. Instead, it engages in autoimmune and pro-inflammatory responses, which favor tissue damage and the acquisition of a low-grade systemic chronic inflammation, known as inflammaging, increasing the risk of many age-related diseases^1^. Thus, the decline of immune function during aging represents a major clinical challenge in many disease conditions including autoimmune diseases, infectious diseases, cancer and neurodegenerative and cardiovascular disorders^2–4^. The use of single-cell RNA sequencing (scRNA-seq) has allowed a deeper characterization of the different subsets of CD4^+^ T cells that accumulate during aging. CD4^+^ age-associated T cells (TAAs) include effector/memory T cells; a population of regulatory T (T_reg_) cells expressing activation genes (denoted as activated T_reg_ cells or aT_reg_ cells); cells with an exhaustion signature (denoted as exhausted); and cells overexpressing genes associated with cytotoxicity that have previously been described in the context of viral infections and cancer as CD4^+^ cytotoxic T cells (denoted as cytotoxic)^5^.

T_reg_ cells, a subtype of CD4^+^ T cells characterized by the expression of the forkhead box transcription factor (FOXP3), are required to maintain immune homeostasis and avoid excessive tissue damage preventing inflammatory and autoimmune diseases^6^. Understanding the molecular mechanisms behind age-associated changes in the T_reg_ compartment is critical to understand the deterioration of the immune system during aging and the consequences for immunosenescence and inflammaging. T_reg_ cells in aged mice are augmented in lymphoid organs (for example, spleen and lymph nodes)^7–9^ and other nonlymphoid tissues such as the visceral fat and lungs^10^, whereas they are reduced in muscle^11^. T_reg_ cells also undergo molecular changes with aging, becoming more dependent on IL-15 signaling owing to a lower expression of the IL-2 receptor CD25, while exhibiting enhanced levels of CD122 (IL-2/IL-15 receptor chain β)^12,13^. Moreover, they acquire a memory-like profile during aging, characterized by the downregulation of CD62L and the upregulation of CD44 and CD69 (refs. ^14,15^). These aged effector–memory T_reg_ cells display increased expression of canonical pro-inflammatory transcription factors such as T-BET and RORγT, and pro-inflammatory cytokines such as IFN-γ or IL-17A^16^. T_reg_ cells with pro-inflammatory phenotypes have been denoted as fragile T_reg_ cells in the context of cancer, although its function in antitumoral responses is still debated^17,18^.

Killer cell lectin-like receptor subfamily G, member 1 (KLRG1) is a co-inhibitory receptor expressed by natural killer (NK) cells and antigen-experienced T cells. The expression of KLRG1 increases dramatically with age in human blood samples^19^. In mice, KLRG1 has been used to identify memory precursor cells from effector T cells. In acute viral infection models, KLRG1 distinguishes short-lived effector CD8^+^ T cells (KLRG1^hi^) and memory precursor effector CD8^+^ T cells (KLRG1^lo^)^20,21^. Importantly, KLRG1 identifies senescent CD8^+^ T cells in humans and mice, as KLRG1^+^CD8^+^ T cells exhibit reduced cytokine production and senescence characteristics such as a reduced proliferative capacity^19,22–24^. Regarding CD4^+^ T cells, KLRG1 also identifies terminally differentiated cells^25^. Specifically in T_reg_ cells, KLRG1 is considered a marker of effector suppressive T_reg_ cells that display increased levels of activation markers (CD69, CD44, CD103 and CD39) and enhanced production of IL-10 (ref. ^26^).

Mitochondria are the key controller of cellular metabolism, but also play a crucial role as signaling hubs for inflammation and cell death. Mitochondrial function declines with age in different cell types and tissues^27^, including T cells^28,29^, affecting the mitochondrial ATP generation as well as reactive oxygen species (ROS) production and cellular signaling^30^. In addition, mitochondrial dysfunction is sufficient to drive cellular senescence in fibroblasts^31,32^. In contrast to conventional T cells, which engage glycolysis during immune response, T_reg_ cells preferentially use mitochondrial respiration as source of ATP^33^ and the genetic induction of T_reg_-specific mitochondrial dysfunction leads to premature death by uncontrolled inflammation^34,35^. Here, by using multiparametic spectral flow cytometry, we investigate the specific subpopulations of T cells that accumulate mitochondrial alterations during aging. Among them, we identify a population of T_reg_ cells characterized by the expression of KLRG1 (kT_reg_ cells) and FOXP3 that displays senescence features such as nuclear DNA damage. Although the suppressive capacity of kT_reg_ cells is maintained in vitro, it is compromised in vivo. kT_reg_ cells produce pro-inflammatory cytokines and, importantly, are also increased among human peripheral blood mononuclear cells (PBMCs) with age.

## Results

### T cells with mitochondrial alterations accumulate with aging

**Fig. 1:**
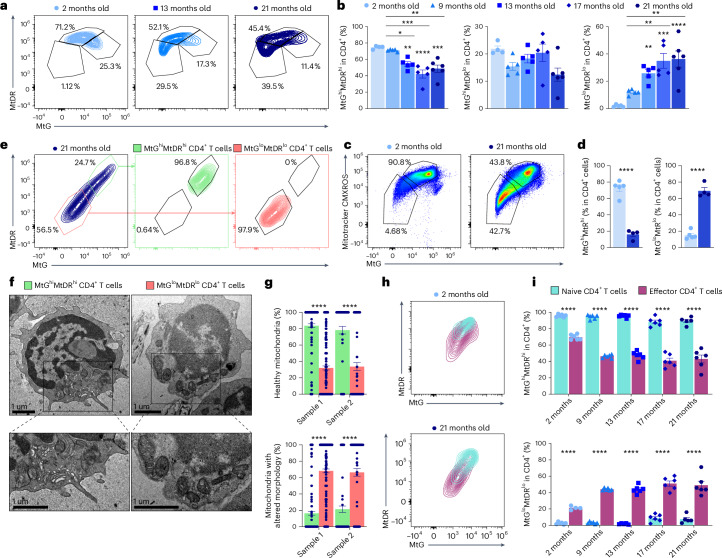
CD4^+^ T cells progressively accumulate altered mitochondria during aging. **a**,**b**, Representative flow cytometry plots showing the simultaneous analysis of MtG and MtDR (**a**) and the percentage of cells with MtG^hi^MtDR^hi^, MtG^hi^MtDR^lo^ and MtG^lo^MtDR^lo^ (**b**) in the circulating CD4^+^ T cells from 2-month-old (*n* = 5), 9-month-old (*n* = 5), 13-month-old (*n* = 5), 17-month-old (*n* = 5) and 21-month-old (*n* = 6) mice. **c**,**d**, Representative flow cytometry plots (**c**) and quantifications (**d**) of the simultaneous analysis of MtG and MitoTracker Red CMXROS in splenic CD4^+^ T cells from young (2-month-old) and aged (21-month-old) mice. **e**, Representative flow cytometry plots showing the sorting strategy of MtG^hi^MtDR^hi^ and MtG^lo^MtDR^lo^ CD4^+^ T cells from old mice. **f**,**g**, Representative images of electron microscopy (**f**) and quantifications (**g**) of healthy and morphologically altered mitochondria in MtG^hi^MtDR^hi^ (green) and MtG^lo^MtDR^lo^ (red) splenic CD4^+^ T cells from old mice (*n* = 90 and 300 cells from 2 different mice). **h**,**i**, Representative flow cytometry plots (**h**) and quantifications (**i**) of MtG^hi^MtDR^hi^ and MtG^lo^MtDR^lo^ cells in circulating naive (CD62L^hi^CD44^lo^, blue) and effector (CD62L^lo^CD44^hi^, purple) CD4^+^ T cells from 2-, 9-, 13-, 17- and 21-month-old mice (*n* = 6 mice per group). Each dot represents an individual mouse. Data are presented as mean values ± s.e.m. Statistical analysis was performed using one-way ANOVA with with post hoc Tukey’s correction (**b**), two-tailed unpaired Student’s *t*-test (**d** and **i**: MtDR^hi^MtG^hi^ 2 months, 9 months, 17 months, 21 months; MtDR^lo^MtG^lo^ 2 months, 9 months, 21 months), two-tailed Welch’s *t*-test (**i**: MtDR^hi^MtG^hi^ 13 months; MtDR^lo^MtG^lo^ 13 months, 17 months) or Mann–Whitney *U* test (**g**). *P < 0.05; **P < 0.01; ***P < 0.001; ****P < 0.0001. Exact *P* values and additional statistical parameters can be found in the source data. Source data

**Extended Data Fig. 1:**
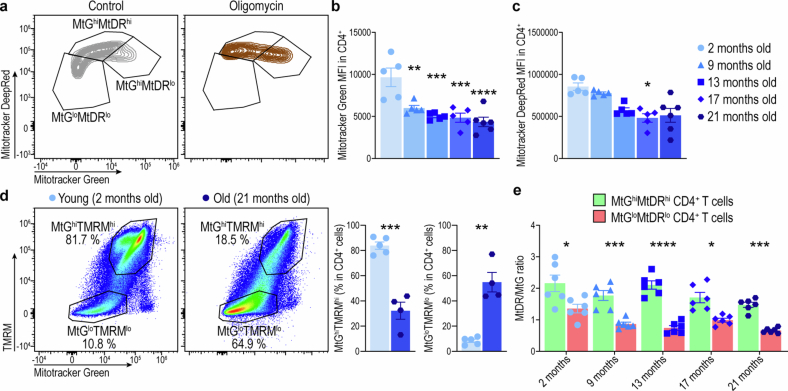
CD4^+^ T cells progressively lose mitochondrial mass and mitochondrial membrane potential during aging. (**a**) Representative plots of mitochondrial mass and mitochondrial membrane potential analyzed by flow cytometry in CD4^+^ T cells treated or not treated with oligomycin. Cells were gated as MtG^hi^MtDR^hi^, MtG^hi^MtDR^lo^ and MtG^lo^MtDR^lo^. Mitochondrial mass analyzed as gMFI of MtG (**b**) and mitochondrial membrane potential analyzed as gMFI of MtDR (**c**) measured by flow cytometry in circulating CD4^+^ T cells from mouse from 2- (n = 5), 9- (n = 5), 13- (n = 5), 17- (n = 5) and 21- (n = 6) month-old mice. (**d**) Representative flow cytometry plots and quantifications of the simultaneous analysis of MtG and TMRM in splenic CD4^+^ T cells from young (2-month-old, n = 5) and old (21-month-old, n = 4) mice. (**e**) Quantification of the MtDR/MtG ratio in MtG^hi^MtDR^hi^ and MtG^lo^MtDR^lo^ CD4^+^ T cells from mice at different ages (n = 6 mice per group). gMFI: Geometric Mean Fluorescence Intensity; MtG: Mitotracker Green; MtDR: Mitotracker DeepRed. Each dot represents an individual mouse. Data are presented as mean values ± SEM. Statistical analysis was performed using one-way ANOVA with post hoc Tukey’s correction (**b**), Kruskal-Wallis test with post hoc Dunn’s correction (**c**), two-tailed Student’s t test [(**d**): TMRM^hi^MtG^hi^; (**e**)] or two-tailed unpaired Welch’s t test [(**d**): TMRM^lo^MtG^lo^] (**b**). *P < 0.05; **P < 0.01; ***P < 0.001; ****P < 0.0001. Exact *P* values and additional statistical parameters can be found in the source data. Source data

To investigate age-associated mitochondrial dysfunction in T cells, we examined mitochondrial mass and mitochondrial membrane potential (MMP) by flow cytometry in circulating CD4^+^ T cells from C57BL/6 mice at different ages ranging from 2 to 21 months old by combining MitoTracker Green (MtG) and MitoTracker DeepRed (MtDR) staining. As a control, we used oligomycin that, by inhibiting the proton ATPase, increased the MMP and therefore the MtDR signal (Extended Data Fig. 1a). Both the MtG and MtDR signals declined during aging in circulating CD4^+^ T cells (Extended Data Fig. 1b,c), suggesting that the proportion of T cells with mitochondrial alterations increases in aged mice (Fig. 1a,b). We obtained similar results using two additional mitochondrial probes: MitoTracker Red CM-X-ROS (Fig. 1c,d) and tetramethylrhodamine methyl ester (TMRM) (Extended Data Fig. 1d). Interestingly, MtG^lo^MtDR^lo^ cells, which accumulate with aging, showed a reduced MtDR/MtG ratio, suggesting mitochondrial defects (Extended Data Fig. 1e). To confirm that MtG^lo^MtDR^lo^ cells have altered mitochondria, we sorted MtG^hi^MtDR^hi^ and MtG^lo^MtDR^lo^ CD4^+^ T cells from aged mice and performed electron microscopy (Fig. 1e,f). MtG^lo^MtDR^lo^ cells showed an increased percentage of mitochondria with altered morphology^36^ identified as round, small and with unstructured or even lost mitochondria cristae (Fig. 1f,g).

Because the T cell subsets dramatically change with age and the most notable changes are the loss of naive T cell subsets and the increase in the effector memory pool^37^, we assessed mitochondrial probes in naïve (CD62L^hi^CD44^lo^) and effector–memory (CD62L^lo^CD44^hi^) CD4^+^ T cells in mice at different ages. We observed that, even in old mice, naive T cells were MtG^hi^MtDR^hi^, while effector–memory cells were characterized by MtG^lo^MtDR^lo^, even in younger mice (Fig. 1h,i).

### KLRG1 identifies age-associated regulatory T cells

**Fig. 2:**
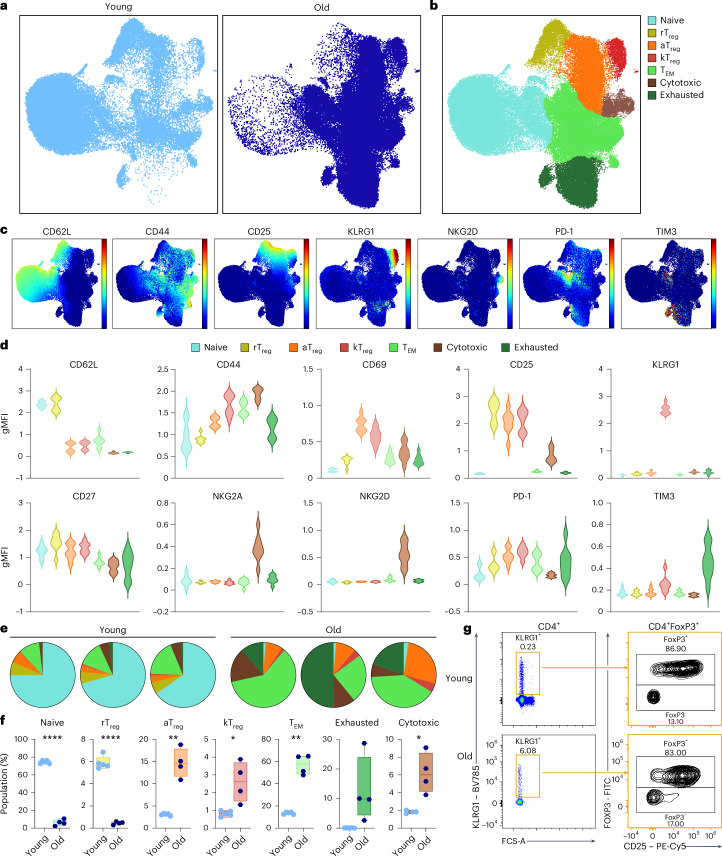
Multiparametric spectral flow cytometry identifies a new age-associated T_reg_ subset. **a**, UMAP representation of splenic CD4^+^ T cells from young (left) and old mice (right) analyzed by spectral flow cytometry. **b**, UMAP with Cluster-X overlay showing the distribution of the different clusters of CD4^+^ T cells identified by spectral flow cytometry (*n* = 4 mice per group). **c**, UMAP representation of the expression levels of representative markers used to identify TAAs by flow cytometry. **d**, The distribution of the gMFI of representative markers used to identify TAAs in CD4^+^ T cell subpopulations: naive (blue), rT_reg_ (yellow), aT_reg_ (orange), kT_reg_ (red), T_EM_ (light green), cytotoxic (brown) and exhausted (dark green) (*n* = 4 mice per group). **e**,**f**, Representative pie charts (**e**) and box plots (**f**) comparing the percentage of cells belonging to each T cell subset in young (2-month-old, *n* = 4) and old (22-month-old, *n* = 5) mice. **g**, The gating strategy to identify FOXP3^+^ cells within the kT_reg_ cells in young (2-month-old) and old (22-month-old) mice (*n* = 3 per group). gMFI, geometric mean fluorescence intensity. Each dot represents an individual mouse. Box-and-whisker plots show the median, the maximum, the minimum and the 25th and 75th percentiles. Statistical analysis was performed using two-tailed unpaired Student’s *t*-test (**f**: naive) or two-tailed unpaired Welch’s *t*-test (**f**: rT_reg_, aT_reg_, kT_reg_, T_EM_, exhausted and cytotoxic). *P < 0.05, **P < 0.01, ***P < 0.001, ****P < 0.0001. Exact *P* values and additional statistical parameters can be found in the source data. Source data

**Extended Data Fig. 2:**
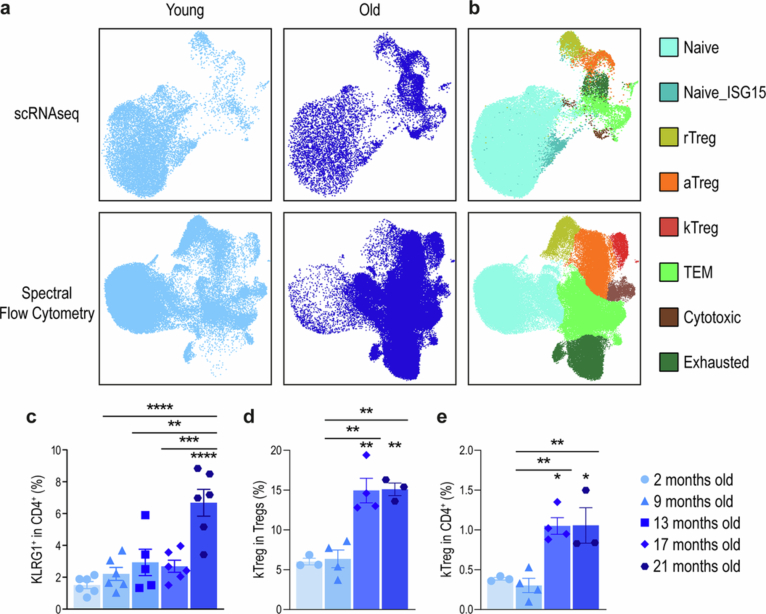
Identification of age-associated T cells by scRNAseq and by spectral flow cytometry. (**a**) UMAP representation of CD4^+^ T cells from young (left) and old mice (right) analyzed by scRNAseq (top) or spectral flow cytometry (bottom). (**b**) UMAP with clustering overlay showing the distribution of the different clusters of CD4^+^ T cells identified by scRNAseq or by spectral flow cytometry: naive (blue), naïve_ISG15 (dark blue), rTregs (yellow), aTregs (orange), kTregs (red), TEM (light green), cytotoxic (brown) and exhausted (dark green) (n = 4 mice per group). (**c**) Percentage of circulating KLRG1^+^ cells in CD4^+^ T cells from 2- (n = 6), 9- (n = 6), 13- (n = 5), 17- (n = 6) and 21- (n = 6) month-old mice assessed by flow cytometry. (**d-e**) Percentage of splenic KLRG1^+^ cells in CD4^+^FOXP3^+^CD25^+^ T cells (**d**) or in CD4^+^ T cells (**e**) from 2- (n = 3), 9- (n = 4), 17- (n = 4) and 21- (n = 4) month-old mice. Each dot represents an individual mouse. Data are presented as mean values ± SEM. Statistical analysis was performed using one-way ANOVA with post hoc Tukey’s correction [(**c**), (**d**), (**e**)]. *P < 0.05; **P < 0.01; ***P < 0.001; ****P < 0.0001. Exact *P* values and additional statistical parameters can be found in the source data. Source data

To assess whether age-associated mitochondrial dysfunction equally affects all TAA subsets, we developed a multiparametric staining panel to identify the different age-associated CD4^+^ T cell subsets. Our panel included surface markers based on gene expression of surface proteins identified by scRNA-seq^5,38^, complemented with known markers for immunosenescence, such as KLRG1 (ref. ^19^). In previous scRNA-seq experiments, splenic T cells from young mice were mainly classified as naive, naive_ISG15 and rT_reg_ cells, whereas T cells from old mice were characterized by the accumulation of TAAs such as aT_reg_, effector memory T (T_EM_) cells, exhausted and cytotoxic T cells^5^. Unbiased clusterization of spectral flow cytometry analysis of splenic CD4^+^ T cells from young (2-month-old) and old (21-month-old) mice resulted in seven subpopulations of T cells (Fig. 2a,b). Naive T cells were characterized by the expression of the adhesion molecule CD62L and the absence of the activation marker CD44. Three subsets of T_reg_ cells were identified by the expression of CD25 and discriminated by different expression levels of CD62L, CD44 and KLRG1. The other CD4^+^ T cell clusters presented marks of differentiation (CD62L^lo^ and CD44^hi^) and were identified as cytotoxic based on the expression of NK-like markers, such as NKG2A and NKG2D, or as exhausted, based on the expression of classical exhaustion markers such as PD-1 and TIM3 (Fig. 2c,d). Young mice mostly displayed naive and rT_reg_ cells, while aT_reg_, kT_reg_, T_EM_, cytotoxic and exhausted cells were increased in spleen from old mice (Fig. 2e,f). Importantly, this panel discriminated six out of the seven subpopulations previously defined by scRNA-seq analyses; however, because the panel is based on surface markers, we could not identify the ISG15_naive T cell subpopulation (Extended Data Fig. 2a,b). Our analysis identified a TAA subset characterized by the expression of KLRG1 (Fig. 2b and Extended Data Fig. 2a,b). In fact, circulating KLRG1^+^ CD4^+^ T cells progressively accumulated with aging (Extended Data Fig. 2c). Notably, these cells co-expressed KLRG1 and CD25, suggesting that KLRG1^+^ CD4^+^ T cells are probably a subpopulation of T_reg_ cells (Fig. 2c,d). To confirm whether these CD25^+^ KLRG1^+^ cells were T_reg_ cells, we combined the panel with the intracellular marker FOXP3 and we found that most of KLRG1^+^ cells expressed CD25 and FOXP3 (83–87%) (denoted as kT_reg_ cells), supporting their T_reg_ identity (Fig. 2g). The percentages of splenic kT_reg_ cells assessed by spectral flow cytometry gradually increased with age within both the T_reg_ compartment and CD4^+^ T cells (Extended Data Fig. 2d,e).

**Extended Data Fig. 3:**
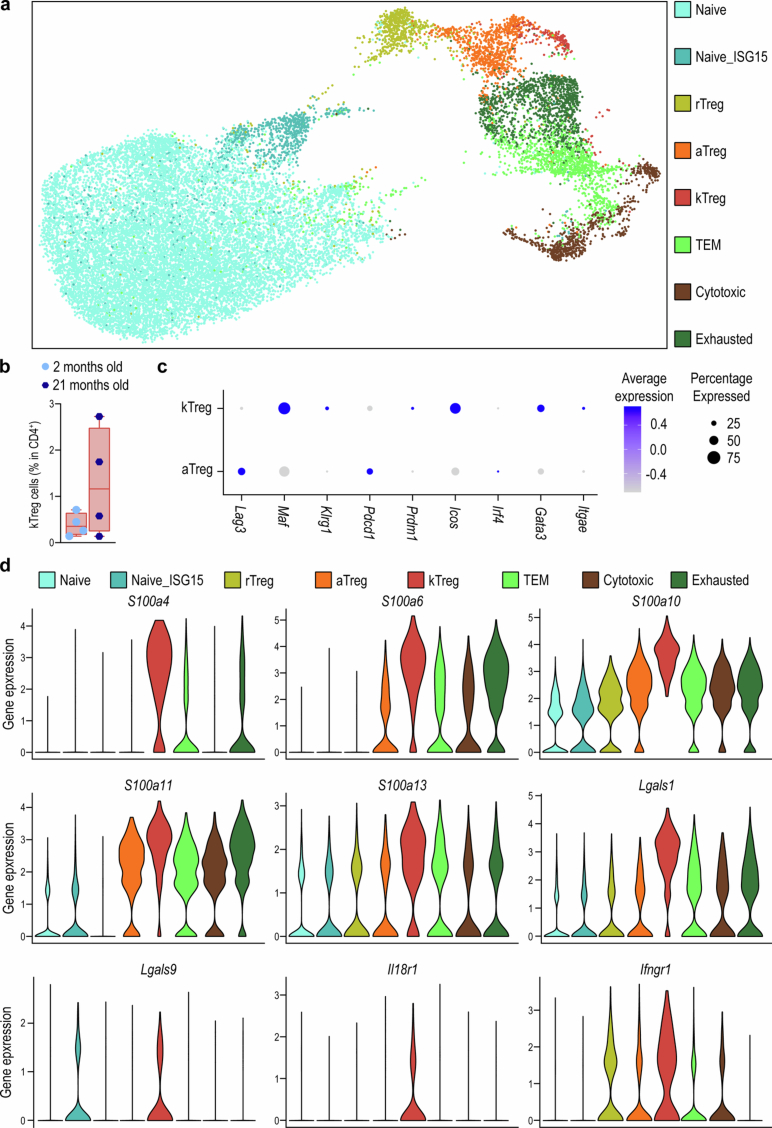
Reanalysis of publicly available scRNAseq data identifies kTregs with a pro-inflammatory signature. (**a**) UMAP representation and clustering of a new analysis of publicly available scRNAseq data from CD4^+^ T cells from young and old mice. Clusters are colored as naïve (blue), rTregs (yellow), aTregs (orange), kTregs (red), T_EM_ (light green), cytotoxic (brown) and exhausted (dark green). (**b**) Quantification of the percentage of kTreg cells in young and old mice (n = 4 mice per group). (**c**) Comparative expression of genes related to Treg function in aTreg and kTreg (n = 4 mice per group). (**d**) Violin plots showing the expression of several pro-inflammatory markers in each cluster of CD4^+^ T cells (n = 4 mice per group). Each dot represents an individual mouse. Box-and-whisker plots show the median, the maximum, the minimum and 25th and 75th percentiles. Statistical analysis was performed using two-tailed unpaired Welch’s t test (**b**). Exact *P* values and additional statistical parameters can be found in the source data. Source data

In addition, we reanalyzed existing and publicly available scRNA-seq data from young and old mice to separate the novel kT_reg_ cluster^5^. By increasing the depth of the clusterization, we obtained an additional cluster, which accumulated during aging, within the aT_reg_ subset (Extended Data Fig. 3a,b). Interestingly, the most determinant genes of this cluster were reminiscent of kT_reg_ cells (Supplementary Table 1). These cells showed increased gene expression of *Klrg1* and altered expression of genes related to T_reg_ function compared with aT_reg_ cells, such as *Icos* and *Maf* (Extended Data Fig. 3c). We also found that kT_reg_ cells expressed higher levels of inflammatory genes such as those belonging to the s100a family (including *S100a4*, *S100a6*, *S100a10*, *S100a11* and *S100a13*) and inflammation-related proteins such as galectins (*Lgals1* and *Lgals9*), IL-18 receptor (*Il18r*) and IFN-γ receptor (*Ifngr1*) (Extended Data Fig. 3d).

**Fig. 3:**
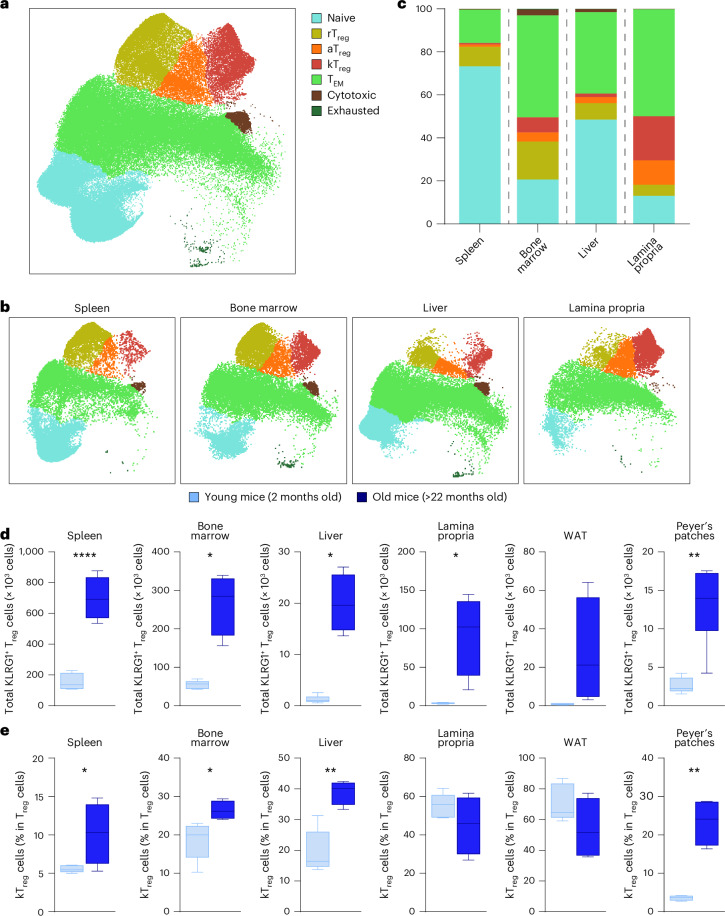
kT_reg_ cells accumulate peripheral tissues during aging and are preferentially located in the colonic lamina propria. **a**, UMAP with Cluster-X overlay showing the distribution of the different clusters of CD4^+^ T cells from spleen, bone marrow, liver and colonic lamina propria of young mice analyzed by spectral flow cytometry. **b**, UMAP with Cluster-X overlay showing the distribution of the different clusters of CD4^+^ T cells from young mice in different tissues (*n* = 5). **c**, Representative bar plots showing the percentage of cells belonging to each T cell subset in spleen, bone marrow, liver and colonic lamina propria from young mice (*n* = 5). **d**,**e**, Quantifications of absolute numbers of kT_reg_ cells (**d**) and the percentage of kT_reg_ cells among T_reg_ cells (**e**) in different tissues in young (2-month-old, *n* = 5) and old (22-month-old, *n* = 4) mice. WAT, white adipose tissue. Box-and-whisker plots show the median, the maximum, the minimum and the 25th and 75th percentiles. Statistical analysis was performed using two-tailed unpaired Student’s *t*-test (**d**: spleen and WAT; **e**: bone marrow, liver, lamina propria and WAT) or two-tailed Welch’s *t*-test (**d**: bone marrow, lamina propria and Peyer’s patches; **e**: spleen and Peyer’s patches]. *P < 0.05, **P < 0.01, ***P < 0.001, ****P < 0.0001. Exact *P* values and additional statistical parameters can be found in the source data. Source data

Then, we investigated the tissue distribution of kT_reg_ cells. To map their preferential location in the different tissues, we used the multiparametric flow cytometry panel to analyze T cells from different tissues including spleen, bone marrow, liver, colonic lamina propria, white adipose tissue and Peyer’s patches from young and old mice. kT_reg_ cells were found in all these tissues (Fig. 3a), and interestingly, the highest percentage of kT_reg_ cells was observed in the colonic lamina propria (Fig. 3b,c). Importantly, the absolute numbers and the percentage of kT_reg_ cells were increased in different tissues during aging (Fig. 3d) and their percentage was increased in spleen, bone marrow, liver and Peyer’s patches but not in lamina propria and white adipose tissue (Fig. 3e). Altogether, we have developed a multiparametric flow cytometry method that allows the identification of TAA subsets in both lymphoid and nonlymphoid organs. Using this strategy, we have identified a subset of T_reg_ cells characterized by the expression of KLRG1 that accumulates with aging.

### KLRG1^+^ T_reg_ cells show mitochondrial alteration and senescence features

**Fig. 4:**
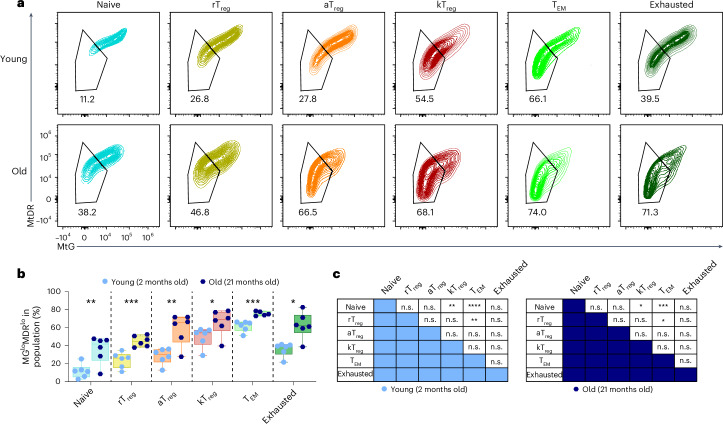
TAAs have different predisposition to mitochondrial perturbations. **a**, Representative flow cytometry plots showing the simultaneous analysis of MtG and MtDR in the different subsets of CD4^+^ T cells from 2- and 21-month-old mice: naive (blue), rT_reg_ cells (yellow), aT_reg_ cells (orange), kT_reg_ cells (red), T_EM_ (light green), cytotoxic (brown) and exhausted (dark green) (*n* = 6 mice per group). **b**, A comparison of the percentage of MtG^lo^MtDR^lo^ cells in each cluster of CD4^+^ T cells in young (2-month-old) and old (21-month-old) mice (*n* = 6 mice per group). **c**, A statistical comparison of the percentage of MtG^lo^MtDR^lo^ cells in the different clusters in young or old mice (*n* = 6 mice per group). Each dot represents an individual mouse. Data are presented as mean values ± s.e.m. Box-and-whisker plots show the median, the maximum, the minimum and the 25th and 75th percentiles. Statistical analysis was performed using two-tailed unpaired Student’s *t*-test (**b**: naive, rT_reg_, aT_reg_ and T_EM_), Mann–Whitney *U* test (**b**: kT_reg_ and exhausted) or Friedman test with post hoc Dunn’s correction (**c**). n.s., not significant; *P < 0.05, **P < 0.01, ***P < 0.001, ****P < 0.0001. Exact *P* values and additional statistical parameters can be found in the source data. Source data

To evaluate if any of the CD4^+^ T cell subsets are more susceptible to the age-associated mitochondrial dysfunction, we combined our spectral flow cytometry panel together with the assessment of mitochondrial probes in young and old mice. Old CD4^+^ T cells showed an increased proportion of MtG^lo^MtDR^lo^ cells in all subsets compared with young CD4^+^ T cells (Fig. 4a,b). Strikingly, even within the same mouse, there is a remarkable difference in the percentage of MtG^lo^MtDR^lo^ in the distinct T cell subsets. While the naive cluster mainly exhibits low frequencies of MtG^lo^MtDR^lo^ cells, T_EM_ cells displayed the major accumulation of MtG^lo^MtDR^lo^ cells in both young and old mice. Among T_reg_ cells, kT_reg_ cells showed an increased percentage of MtG^lo^MtDR^lo^ cells compared with other T_reg_ subsets in both young and old mice (Fig. 4a,c), suggesting that kT_reg_ cells are terminally differentiated T_reg_ cells with mitochondrial alterations.

**Fig. 5:**
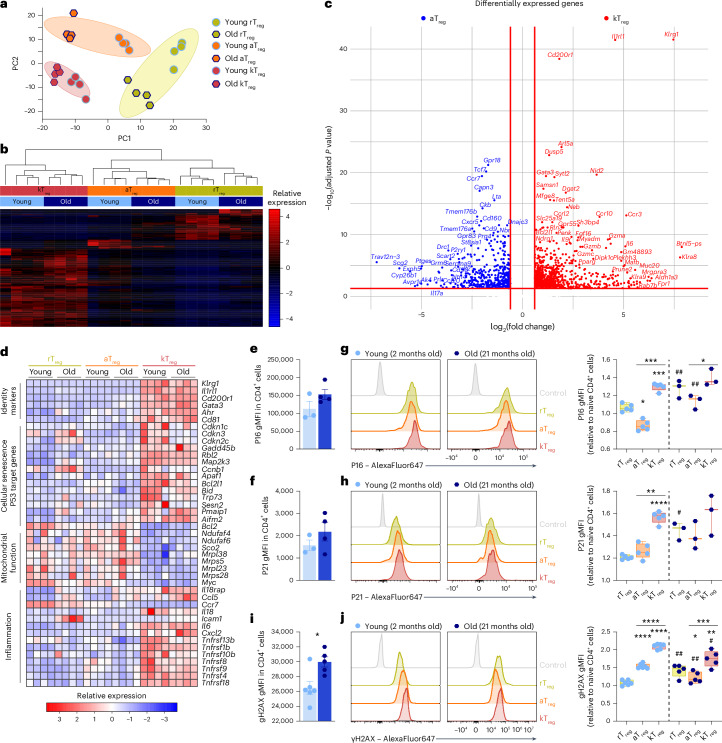
KLRG1 identifies T_reg_ cells with senescence features. **a**–**d**, RNA sequencing of rT_reg_, aT_reg_ and kT_reg_ cells from young and old mice: PCA (**a**) and clusterization (**b**); volcano plot showing the DEGs between aT_reg_ cells and kT_reg_ cells (**c**); heat map of selected DEGs illustrating genes characteristic of identity, cellular senescence, P53 signaling, mitochondrial function and inflammation (**d**) (*n* = 4 per group). **e**,**f**, Quantification of the expression of P16 (**e**) and P21 (**f**) by flow cytometry in splenic CD4^+^ T cells from young (2 months old, *n* = 4) and old (21 months old, *n* = 3) mice measured as gMFI. **g**,**h**, Representative histograms and quantifications of the expression of P16 (**g**) and P21 (**h**) by flow cytometry in the different subsets of splenic T_reg_ cells from young (*n* = 4) and old (*n* = 3) mice measured as gMFI: rT_reg_ cells (yellow), aT_reg_ cells (orange) and kT_reg_ cells (red). **i**, Quantification of γH2AX gMFI in CD4^+^ T cells from young and old mice (*n* = 5 per group). **j**, Representative histogram and quantification of γH2AX by flow cytometry in the different subsets of T_reg_ cells from young and old mice measured as gMFI (*n* = 5 per group). gMFI values are relative to the gMFI in naive CD4^+^ T cells. DEGs, differentially expressed genes; gMFI, geometric mean fluorescence intensity. Each dot represents an individual mouse. Data are presented as mean values ± s.e.m. Statistical analysis between young and old mice was performed using two-tailed unpaired Student’s *t*-test (**e**–**h**: aT_reg_; **i** and **j**) or two-tailed Welch’s *t*-test (**h**: rT_reg_ and kT_reg_). Statistical analysis between subsets within the same animal was performed using RM one-way ANOVA with with post hoc Tukey’s correction (**g**, **h** and **j**). Asterisks refers to statistic comparisons between rT_reg_, aT_reg_ and kT_reg_ in the same age group; the number signs refer to statistic comparisons between young and old mice. *P < 0.05, **P < 0.01, ***P < 0.001, ****P < 0.0001. Exact *P* values and additional statistical parameters can be found in the source data. Source data

To better characterize the different T_reg_ subpopulations, we compared the transcriptomic fingerprint of rT_reg_, aT_reg_ and kT_reg_ cells isolated from young (2-month-old) and old (26-month-old) mice by bulk RNA-seq. This transcriptomic dataset allowed us to improve the resolution of the kT_reg_ cell expression profile. The clusterization grouped the samples by their identity rather than the age of the mice (Fig. 5a,b). We then analyzed the differentially expressed genes between the two age-associated T_reg_ subsets, aT_reg_ and kT_reg_. We found *Klrg1*, *Il1rl1* and *Cd200r1* as the most significantly increased genes in kT_reg_ cells. Other genes upregulated in kT_reg_ cells included *Foxp3*, *Gata3*, *Ahr*, NK-related genes (*Klra8*), granzymes (*Gzmb*, *Gzma* and *Gzmc*) and chemokine receptors (*Ccr3* and *Ccr10*) (Fig. 5c). The pathway analysis showed that kT_reg_ cells exhibit increased expression of cellular senescence genes and P53 target genes, decreased expression of genes related to mitochondrial function and increased expression of inflammatory genes (Fig. 5d).

Given that a mitochondrial dysfunction is a trigger of cellular senescence^31^, and that the transcriptional fingerprint of kT_reg_ cells showed increased expression of senescence-related genes, we interrogated whether kT_reg_ cells display other features of cellular senescence beyond mitochondrial alterations. We analyzed the expression of the cell cycle regulators P16 and P21, which are increased in CD4^+^ T cells during aging (Fig. 5e,f). We compared the three different subsets of T_reg_ cells and found that kT_reg_ cells displayed the highest expression of P16 and P21 (Fig. 5g,h). Moreover, as an additional marker of cellular senescence, we analyzed the genomic DNA damage measured as the phosphorylation of the histone H2AX in the serine 139 (γH2AX), a marker of DNA double strand breaks that is increased in CD4^+^ T cells from old mice (Fig. 5i). Interestingly, kT_reg_ cells also displayed increased DNA damage compared with aT_reg_ cells (Fig. 5j).

### KLRG1^+^ T_reg_ cells express ST2 and are induced by IL-33 in vivo

**Fig. 6:**
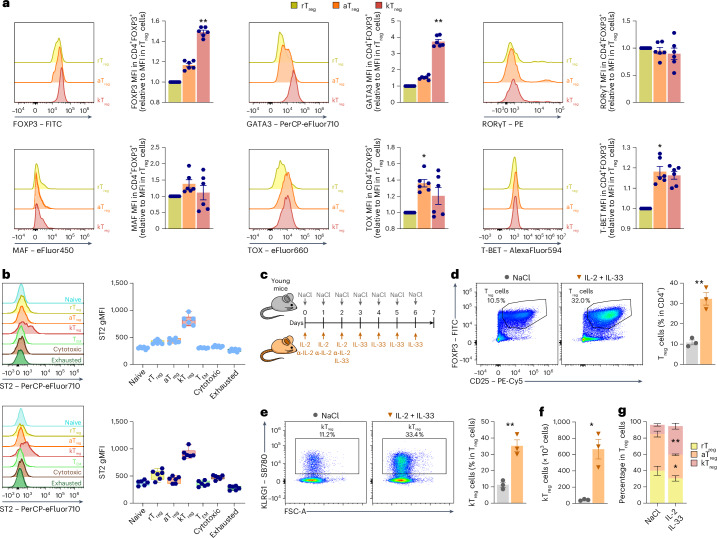
KLRG1^+^ T_reg_ differentiation depends on the IL-33–ST2 axis. **a**, Representative histograms and quantifications of the expression of different transcription factors by flow cytometry in splenic rT_reg_ (yellow), aT_reg_ (orange) and kT_reg_ (red) cells from old (21-month-old) mice measured as gMFI (*n* = 6 mice per group). gMFI values are relative to the MFI in rT_reg_ cells (*n* = 6 per group). **b**, Representative histograms and quantifications of ST2 expression by flow cytometry in the different clusters of splenic CD4^+^ T cells from young (2-month-old) and old (21-month-old) mice: naive (blue), rT_reg_ (yellow), aT_reg_ (orange), kT_reg_ (red), T_EM_ (light green), cytotoxic (brown) and exhausted (dark green) cells measured as gMFI (*n* = 6 mice per group). **c**–**g**, In vivo differentiation of kT_reg_ cells: young control mice (2 months old) were treated with IL-2 + IL-33 or NaCl for 6 days and analyzed on day 7 after injection (*n* = 3 mice per group) (**c**); representative flow cytometry plot and quantification of the proportion of splenic T_reg_ cells in young mice treated with NaCl or IL-2 and IL-33 (**d**); representative flow cytometry plot and quantification of the proportion of KLRG1^+^ T_reg_ cells in young mice treated with NaCl or IL-2 and IL-33 (**e**); quantification of the absolute numbers of KLRG1^+^ T_reg_ cells in young mice treated or not treated with IL-2 and IL-33 (*n* = 3 mice per group) (**f**); quantification of the percentage of each T_reg_ cluster in young mice treated with NaCl or IL-2 and IL-33 (*n* = 3 mice per group) (**g**). gMFI, geometric mean fluorescence intensity. Each dot represents an individual mouse. Data are presented as mean values ± s.e.m. Box-and-whisker plots show the median, the maximum, the minimum and the 25th and 75th percentiles. Statistical analysis was performed using Friedman test with post hoc Dunn’s correction (**a** and **b**: old), RM one-way ANOVA with post hoc Tukey’s correction (**b**: young), two-tailed unpaired Student’s *t*-test (**d**, **e** and **g**) or two-tailed Welch’s *t*-test (**f**). *P < 0.05, **P < 0.01, ***P < 0.001, ****P < 0.0001. Exact *P* values and additional statistical parameters can be found in the source data. Source data

**Extended Data Fig. 4:**
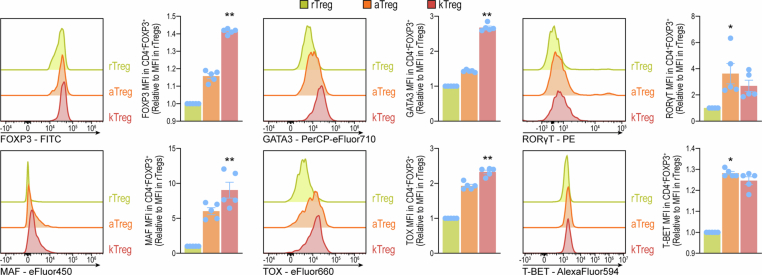
Analysis of the expression of different transcription factors in the Treg subsets. Representative histograms and quantifications of the expression of different transcription factors by flow cytometry in splenic rTreg (yellow), aTreg (orange) and kTregs (red) from young (2-month-old) mice (n = 6 mice per group) measured as gMFI. gMFI values are relative to the MFI in rTregs (n = 6 per group). gMFI: Geometric Mean Fluorescence Intensity. Each dot represents an individual mouse. Data are presented as mean values ± SEM. Statistical analysis was performed using Friedman test with post hoc Dunn’s correction. *P < 0.05; **P < 0.01; ***P < 0.001; ****P < 0.0001. Exact *P* values and additional statistical parameters can be found in the source data. Source data

In light of the increased expression of *Gata3* in kT_reg_ cells (Fig. 5c), and to further characterize the kT_reg_ subset, we compared the expression of different transcription factors related to T cell function by flow cytometry. This analysis revealed that kT_reg_ cells expressed higher levels of FOXP3 compared with the other T_reg_ subsets (Fig. 6a and Extended Data Fig. 4), whereas the levels of RORγT and T-BET were similar to aT_reg_ cells. Importantly, kT_reg_ cells expressed higher levels of GATA3 than aT_reg_ cells in both young and old mice (Fig. 6a and Extended Data Fig. 4), supporting the RNA-seq results. TOX and MAF expression was higher in kT_reg_ cells compared with rT_reg_ and aT_reg_ cells, but this difference was not observed in old mice (Fig. 6a and Extended Data Fig. 4).

GATA3 is essential for the regulatory function of T_reg_ cells^39,40^. Given that GATA3-expressing T_reg_ cells are characterized by the expression of the IL-33 receptor ST2 (refs. ^41,42^), we analyzed the expression of ST2 in the different CD4^+^ T cell clusters. We observed that kT_reg_ cells expressed higher levels of ST2 than the rest of T cell subsets in both young and old mice (Fig. 6b). Based on that, we tried to induce the differentiation of kT_reg_ cells in vivo by administration of IL-33 in young mice. Before IL-33 administration, we injected IL-2, which induces the expansion of T_reg_ cells due to their expression of the IL-2 receptor CD25 (ref. ^43^) (Fig. 6c). Upon this treatment, we found an expansion of T_reg_ cells in the injected mice (Fig. 6c) and an increase in the percentage and absolute number of kT_reg_ cells (Fig. 6e,f). Interestingly, this treatment seems to induce the differentiation of aT_reg_ cells into kT_reg_ cells (Fig. 6g), suggesting that kT_reg_ cells rely on ST2–IL-33 signaling for differentiation.

### KLRG1^+^ T_reg_ cells exhibit a pro-inflammatory phenotype

**Fig. 7:**
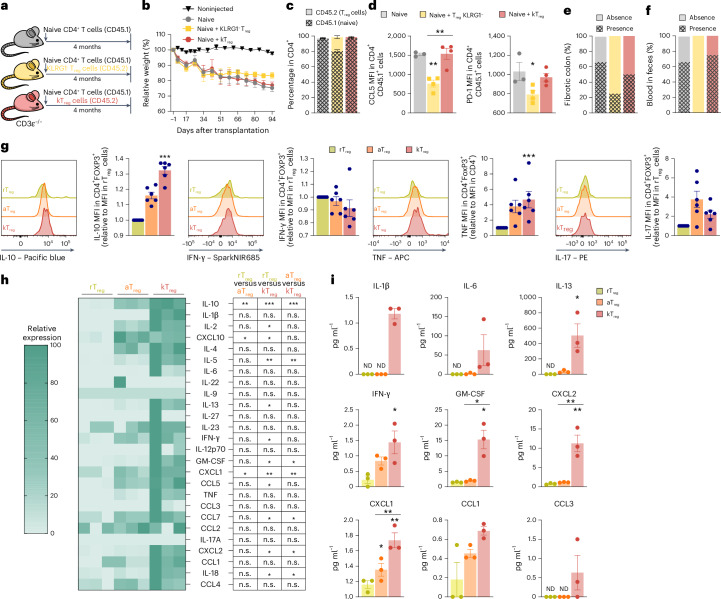
Analysis of the suppressive and pro-inflammatory function of KLRG1^+^ T_reg_ cells. **a**–**f**, In vivo assessment of the suppressive activity of KLRG1^+^ T_reg_ cells. CD3ε^−/−^ T cell-deficient mice were exclusively injected with naive CD45.1 CD4^+^ T cells (*n* = 4) or in combination with either CD45.2 KLRG1^−^ T_reg_ cells (*n* = 5) or CD45.2 KLRG1^+^ T_reg_ cells (*n* = 5) isolated from young mice treated with IL-2 + IL-33. Noninjected CD3ε^−/−^ (*n* = 3) are represented for the sake of comparison. The injected mice were monitored for 4 months and analyzed. **a**, Schematic diagram depicting the in vivo suppression assay. **b**, The body weight of CD3ε^−/−^ mice injected with naive CD4^+^ T cells alone or in combination with KLRG1^−^ or KLRG1^+^ T_reg_ cells. The weight of each animal was normalized to its own weight before the inoculation. **c**, The percentage of transferred CD45.1 naive (striped color) or CD45.2 T_reg_ (flat color) cells analyzed by flow cytometry in CD3ε^−/−^ mice injected with naive and/or T_reg_ cells. **d**, Quantification of the expression of CCL5 and PD-1 by flow cytometry in the injected CD45.1 CD4^+^ T cells measured as gMFI. **e**,**f**, The percentage of mice with fibrotic colon (**e**) or fecal blood (**f**) in mice injected with naive and/or T_reg_ cells. **g**, Representative histograms and quantifications of the expression of different cytokines by flow cytometry in splenic rT_reg_ (yellow), aT_reg_ (orange) and kT_reg_ (red) cells from old (21-month-old) mice measured as gMFI. gMFI values are relative to the MFI in rT_reg_ cells (*n* = 6). **h**,**i**, Analysis of the secretion of different cytokines by the T_reg_ subsets using multiplex. T_reg_ subsets were sorted from young mice injected with IL-2 + IL-33 and incubated during 24 h in 10% FBS complete RPMI medium before the assay (*n* = 3). Heat map (**h**) and statistical comparisons of the secretion of different cytokines by rT_reg_, aT_reg_ and kT_reg_ cells. Heat map values are relative to the maximum of each cytokine. Quantification (**i**) of SASP-related cytokines secreted by rT_reg_, aT_reg_ and kT_reg_ cells. gMFI, geometric mean fluorescence intensity. Each dot represents an individual mouse. Data are presented as mean values ± s.e.m. Statistical analysis was performed using one-way ANOVA with post hoc Tukey’s correction (**c**: CD45.1; **d** and **e**), Krukal–Wallis test with post hoc Dunn’s correction (**c**: CD45.2), Friedman test with post hoc Dunn’s correction (**g**–**i**). n.s., not significant; *P < 0.05, **P < 0.01, ***P < 0.001, ****P < 0.0001. Exact *P* values and additional statistical parameters can be found in the source data. Source data

**Extended Data Fig. 5:**
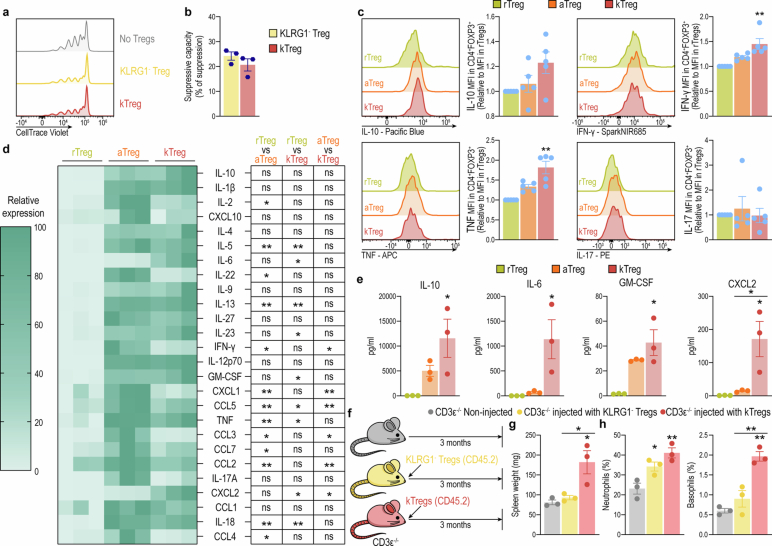
KLRG1^+^ Tregs show a pro-inflammatory phenotype and induce inflammation. (**a-b**) *In vitro* assessment of the suppressive activity of KLRG1^+^ Tregs. KLRG1^-^ or KLRG1^+^ Tregs were sorted from old (22-month-old) and co-cultured with CD45.1 naïve CD4^+^ T responder cells labelled with CellTrace Violet at 2:1 ratio (2 naïve:1 Treg) for 72 h in the presence of α-CD3/α-CD28 (n = 3 mice). (**a**) Representative histogram of the CellTrace Violet signal measured by flow cytometry in CD45.1 CD4^+^ T conventional responder cells after 72 h of culture. (**b**) Quantification of the capacity of KLRG1^-^ and KLRG1^+^ Treg cells to suppress the proliferation of CD45.1 CD4^+^ responder T cells. The percentage of suppression was determined as: 100 − (% of proliferating cells with Tregs)/(% of proliferating cells without Tregs). (**c**) Representative histograms and quantifications of the expression of different cytokines by flow cytometry in splenic rTreg (yellow), aTreg (orange) and kTregs (red) from old (21-month-old) mice measured as gMFI. gMFI values are relative to the MFI in rTregs (n = 6 mice per group). (**d**-**e**) Analysis of the secretion of different cytokines by the Treg subsets in activating conditions using multiplex. Treg subsets were sorted from young mice injected with IL-2 + IL-33 and incubated during 24 h in the presence of α-CD3/α-CD28 before the assay (n = 3 mice). (**d**) Heatmap and statistical comparisons of the secretion of different cytokines by rTreg, aTreg and kTregs. Heatmap values are relative to the maximum of each cytokine. (**e**) Quantification of IL-10, IL-6, GM-CSF and CXCL2 secreted by activated rTreg, aTreg and kTregs. (**f-h**) *In vivo* assessment of the pro-inflammatory activity of KLRG1^+^ Tregs: CD3ε^-/-^ T cell deficient mice were injected with splenic KLRG1^-^ or KLRG1^+^ Tregs isolated from young mice injected with IL-2 + IL-33. The mice were analyzed four months after the adoptive transfer (n = 3 mice per group). (**f**) Schematic diagram depicting the adoptive transfer of kTregs. (**g**) Spleen weight from CD3ε^-/-^ mice non-injected or injected with KLRG1^-^ or KLRG1^+^ T cells. (**h**) Quantification of the percentage of circulating neutrophils or basophils in CD3ε^-/-^ mice non-injected or injected with KLRG1^-^ or KLRG1^+^ T cells. gMFI: Geometric Mean Fluorescence Intensity. Each dot represents an individual mouse. Data are presented as mean values ± SEM. Statistical analysis was performed using two-tailed unpaired Student’s t test (**b**), Friedman test with post hoc Dunn’s correction [(**c**), (**e**): IL-6], RM one-way ANOVA with post hoc Tukey’s correction [(**d**); (**e**): IL10, GM-CSF, CXCL2] or ANOVA with post hoc Tukey’s correction [(**g**), (**h**)]. *P < 0.05; **P < 0.01; ***P < 0.001; ****P < 0.0001. Exact *P* values and additional statistical parameters can be found in the source data. Source data

To investigate the function of kT_reg_ cells, we analyzed the suppressive function of these cells both in vitro and in vivo. First, we isolated T_reg_ cells from old mice, sorted them on the basis of KLRG1 expression and co-cultured them with naive T cells loaded with CellTrace Violet at a 1:2 ratio (1 T_reg_:2 naive). Both KLRG1^+^ and KLGR1^−^ T_reg_ cells showed a similar suppressive capacity in vitro (Extended Data Fig. 5a,b). Next, we performed an in vivo suppression assay by injecting naive CD45.1 CD4^+^ T cells alone or in combination with CD45.2 KLRG1^−^ or KLRG1^+^ T_reg_ cells into T cell-deficient mice (CD3ε^−/−^ CD45.2) (Fig. 7a). While KLRG1^−^ T_reg_ cells were able to restrain the weight loss induced by naive T cells, mice injected with naive T cells together with KLRG1^+^ T_reg_ cells lost weight to a similar extent as mice exclusively injected with naive T cells (Fig. 7b). Four months after the adoptive transfer, CD45.1 cells were more abundant in mice injected with KLRG1^+^ T_reg_ cells (Fig. 7c). In addition, we found that transferred CD45.1 CD4^+^ T cells were more activated when co-transferred with KLRG1^+^ T_reg_ cells, as shown by their increased expression of CCL5 and PD-1, than when co-transferred with KLRG1^−^ T_reg_ cells (Fig. 7d). Importantly, we observed an increased prevalence of fibrotic colon and fecal blood in mice injected with naive T cells alone or together with KLRG1^+^ T_reg_ cells than in mice injected with naive T cells together with KLRG1^−^ T_reg_ cells (Fig. 7e,f). Altogether, these data suggest that the function and/or viability of KLRG1^+^ T_reg_ cells in vivo is compromised.

To further explore the activity of kT_reg_ cells, we assessed their capacity to produce cytokines by ex vivo intracellular flow cytometry. We found that kT_reg_ cells from old mice expressed higher amounts of the anti-inflammatory cytokine IL-10 than the rest of the T_reg_ subsets (Fig. 7g). We found no differences in the production of IL-17 or IFN-γ by kT_reg_ cells compared with aT_reg_ cells, but kT_reg_ cells produced more TNF (Fig. 7g). Similar results were observed when comparing T_reg_ subsets from young mice (Extended Data Fig. 5c). Furthermore, we measured the cytokine secretion of the different T_reg_ subsets. We induced kT_reg_ expansion in vivo by IL-33 administration and sorted rT_reg_, aT_reg_ and kT_reg_ cells. After 24 h in vitro, we assessed the cytokine release by multiplex analysis of the culture media and we observed that, in agreement with our flow cytometry data, kT_reg_ cells secreted higher amounts of IL-10 together with pro-inflammatory cytokines than the other T_reg_ subsets (Fig. 7h). Notably, kT_reg_ cells produced higher levels of senescence-associated secretory phenotype (SASP)-related factors including IL-1β, IL-6, IL-13, IFN-γ, GM-CSF, CXCL2, CXCL1, CCL1 and CCL3 than rT_reg_ or aT_reg_ cells (Fig. 7i). Noteworthy, upon activation, aT_reg_ cells secreted similar or even higher levels of most cytokines than kT_reg_ cells. However, kT_reg_ cells maintained increased production of IL-10, IL-6, GM-CSF and CXCL2 (Extended Data Fig. 5d,e). Finally, to investigate the function of kT_reg_ cells in vivo, we adoptively transferred KLRG1^−^ and KLRG1^+^ T_reg_ cells into CD3ε^−/−^ mice (Extended Data Fig. 5f). In agreement with the increased production of pro-inflammatory cytokines, we found that mice transferred with KLRG1^+^ T_reg_ cells showed splenomegaly together with increased percentages of circulating neutrophils and basophils compared with noninjected or mice injected with KLRG1^−^ T_reg_ cells (Extended Data Fig. 5g,h).

### KLRG1^+^ T_reg_ cells increase in human blood samples during aging

**Fig. 8:**
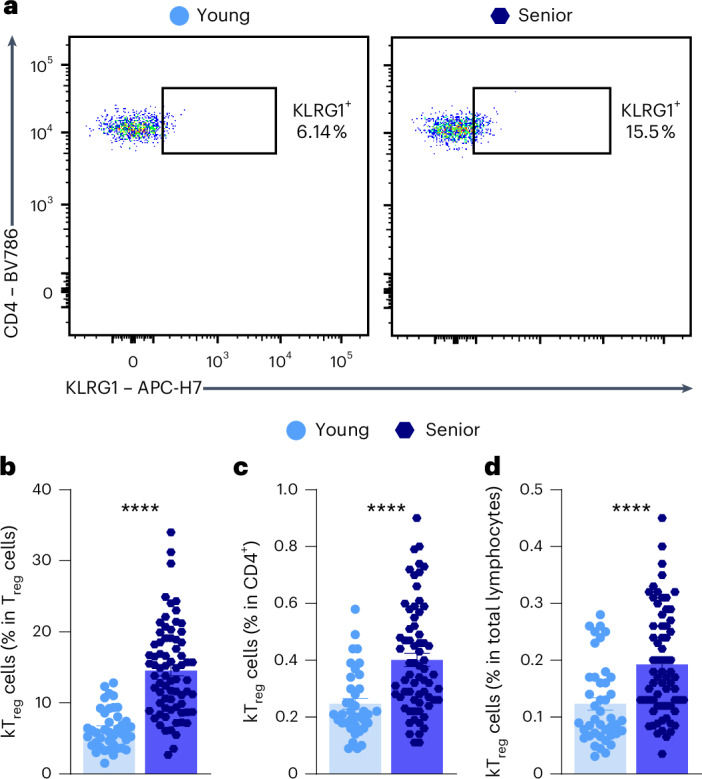
KLRG1^+^ T_reg_ cells are increased in human blood during aging. **a**, Representative flow cytometry plots of the gating of KLRG1^+^ T_reg_ cells in PBMCs samples from young and senior volunteers. **b**–**d**, Quantifications of the percentage of KLRG1^+^ T_reg_ cells among T_reg_ cells (**b**), among CD4^+^ cells (**c**) and among total lymphocytes (**d**) in young (18–25 years old, *n* = 42) and senior (≥55 years old, *n* = 75) individuals by flow cytometry. Each dot represents an individual. Data are presented as mean values ± s.e.m. Statistical analysis was performed using two-tailed unpaired Welch’s *t*-test (**b**) or Mann–Whitney *U* test (**c** and **d**). *P < 0.05, **P < 0.01, ***P < 0.001, ****P < 0.0001. Exact *P* values and additional statistical parameters can be found in the source data. Source data

**Extended Data Fig. 6:**
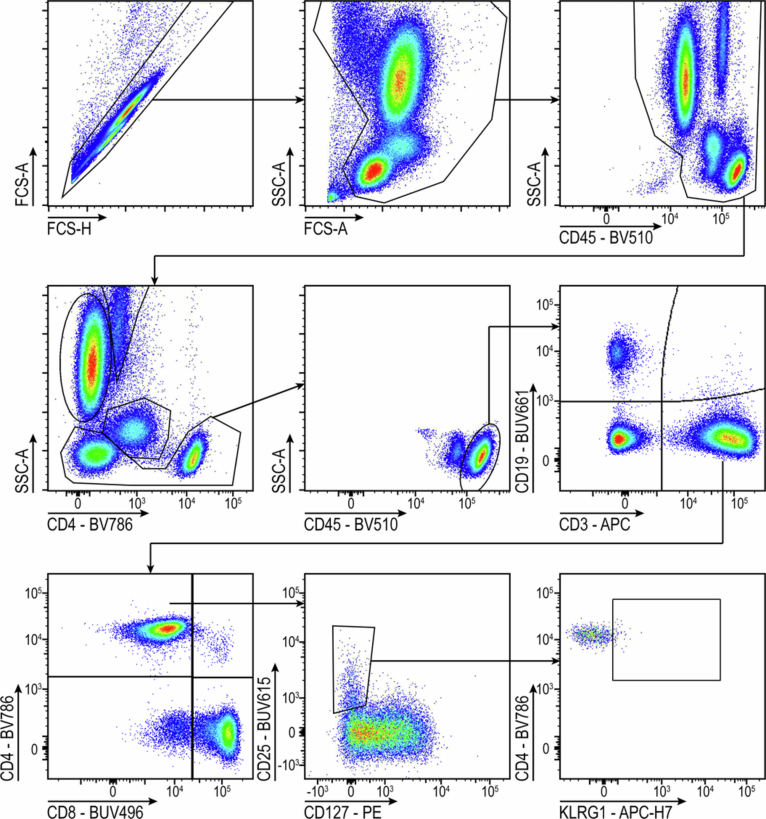
Gating strategy to identify KLRG1^+^ Tregs in human PBMCs. Gating strategy followed to identify KLRG1^+^ Tregs (CD45^+^CD3^+^CD4^+^CD8^-^CD25^+^IL7R^-^KLRG1^+^) in human PBMCs.

We wondered whether the kT_reg_ population is also increased in humans during aging. We used flow cytometry to analyze the presence of kT_reg_ cells in PBMCs from a human cohort of 144 healthy volunteers separated into two groups: young (ranging from 18 to 25 years old) and senior (over 55 years old) (see Extended Data Fig. 6 for the gating strategy). We found an increased frequency of kTregs in the senior population (14.56% of T_reg_ cells) compared with young individuals (6.35% of T_reg_ cells) (Fig. 8).

Altogether, by using deep flow cytometry analysis, we have identified a subset of T_reg_ cells characterized by the expression of KLRG1 and ST2. Besides typical T_reg_ cytokines such as IL-10, kT_reg_ cells produce other SASP-related cytokines and chemokines such as IL-1β, IL-6, IL-13, IFN-γ, GM-CSF, CXCL2, CXCL1, CCL1 and CCL3. kT_reg_ cells also harbor senescence features such as mitochondrial decline, expression of cell cycle inhibitors and DNA damage. These kT_reg_ cells with senescence features are induced in response to IL-33 and accumulate with aging in different tissues, but they are especially abundant in the gut in mice. Importantly, circulating kT_reg_ cells are increased in humans during aging.

## Discussion

T cells play a critical role in recognizing and depleting damage or infected cells and sustaining tissue homeostasis preventing inflammation and tissue deterioration^21^. However, like every cell type in the body, T cells undergo changes as they age, leading to diminished function and compromising tissue homeostasis. Recently, scRNA-seq technology has enabled the decoding of the heterogeneity of age-associated CD4^+^ and CD8^+^ T cells that accumulate over time^5,38^. Using this information, we have developed a multiparametric panel of 20 antibodies, which allows routine discrimination of T cell heterogeneity during aging by spectral flow cytometry. With this strategy, we have mapped the different TAAs that accumulate during aging in different tissues and we have identified KLRG1-expressing T_reg_ cells as a subset accumulating in blood, lymphoid and nonlymphoid tissues during natural aging and harboring aging features, including mitochondrial alterations.

Functional T_reg_ cells are essential to restore tissue homeostasis and to prevent autoimmunity and inflammation. Although it is well established that T_reg_ cells are augmented in several organs during aging in both humans and mice, whether their function is compromised with aging is still a matter of debate. Some authors showed that young and old T_reg_ cells have equivalent suppressive capacity^44,45^, whereas others showed an age-related increase in T_reg_ function linked to increased risk of malignancies and infections in older adults^7,14^. By contrast, there is also evidence of a deterioration of T_reg_ function during aging. Old T_reg_ cells are not able to suppress IL-17^+^ T cells upon chronic inflammation^44^, failing to control hypersensitivity^46^ and producing reduced amounts of IL-10 (ref. ^47^).

KLRG1 is a marker of highly differentiated T cells, and it has been considered as a marker of immunosenescence^21^. In addition, a subset of terminally differentiated T_reg_ cells that express KLRG1 has been identified as short-lived T_reg_ cells expressing higher levels of suppressive (that is, CTLA-4 and IL-10) but also inflammatory molecules^26^ located in nonlymphoid barriers, metabolic tissues or at sites of inflammation^9,48^. For example, KLRG1^+^ T_reg_ cells contribute to insulin resistance^49^ and IFN-γ- and IL-10-secreting KLRG1^+^ T_reg_ cells also infiltrate the central nervous system during experimental autoimmune encephalomyelitis^50^. Using our flow cytometry panel, we mapped the presence of kT_reg_ cells in different tissues including spleen, bone marrow, liver, colonic lamina propria, white adipose tissue, Peyer’s patches and blood during aging. Our results demonstrate that kT_reg_ cells increase during aging in most of the analyzed tissues and that the colonic lamina propria is a preferential location for kT_reg_ cells, reaching 50% of the total T_reg_ pool. Importantly, T_reg_ cells are critical for gut homeostasis^51,52^, which is essential for healthy aging.

Our results also support that kT_reg_ cells express the IL-33 receptor ST2 and that their differentiation depends on IL-33. IL-33 is an interleukin-1-like cytokine that plays a critical role in mucosal immunity, favoring Th2 differentiation^53^. IL-33 is increased in the adipose tissue with aging^54^, and it induces thymic involution and T cell dysfunction^55^. Further research is required to define whether IL-33 levels change in lymphoid tissues or in the circulation during aging or if the differentiation of kT_reg_ cells depends on other factors.

By combining the multiparametric panel with mitochondrial probes, we assessed which of the different T cell subsets that accumulate during aging show mitochondrial dysfunction. We found that, whereas naive T cells are MtG^hi^MtDR^hi^, even in old mice, TAAs show increased percentage of cells with low MtG and MtDR stainings. Within T_reg_ cells, we found that kT_reg_ cells show higher percentage of MtG^lo^MtDR^lo^, indicating that they harbor reduced mitochondrial mass and MMP. The MMP is an important indicator of mitochondrial integrity and function. During the mitochondrial respiration, electrons are ejected from the mitochondrial matrix to the intermembrane space to generate an electrochemical gradient. An optimal MMP is required for most of the mitochondrial functions including the generation of ATP, the uptake of ions, the synthesis of iron–sulfur clusters and others. When the MMP is not recovered, unpolarized mitochondria are degraded by mitophagy^56^. The simultaneous assessment of mitochondrial mass and MMP by flow cytometry have been extensively used to characterize the health status of mitochondria in different tissues, including T cells, under different conditions such as tumor environment^57–60^. Nevertheless, changes in the molecular composition of mitochondria, particularly in cardiolipins, lead to a reduction in the binding of MitoTracker probes in intraepithelial lymphocytes that, besides their low signal of MtG and MtDR, showed a similar mitochondria number on electron microscopy images^61^. Importantly, a reduction in the staining of these probes could also be explained by changes in the plasma membrane potential or by the expression of multidrug resistance transporters that expel the fluorescent probes^62^. To confirm that the changes that we observed in MtG and MDR stating are related to mitochondria, we sorted MtG^hi^MtDR^hi^ and MtG^lo^MtDR^lo^ CD4^+^ T cells and assessed mitochondrial morphology by electron microscopy. Electron microscopy analysis shows that MtG^lo^MtDR^lo^ cells have mitochondrial changes including signs of cristae remodeling, supporting that MtG^lo^MtDR^lo^ T cells have mitochondrial alterations. However, additional research is needed to determine whether the MtG^lo^MtDR^lo^ T cell population also has structural or biochemical changes in the composition of mitochondria, in the plasma membrane potential and/or in the expression of multidrug transporters that could explain the dramatic reduction in MtG and MtDR staining. Importantly, beyond the key role of mitochondrial metabolism for the suppressive function of T_reg_ cells^35^, a proper mitochondrial metabolism of T_reg_ cells is essential for maintaining liver homeostasis during aging. Deleting Altre, a long noncoding RNA found in T_reg_ cells and upregulated with aging, leads to mitochondrial dysfunction, causing liver inflammation, fibrosis and cancer in aged mice^63^. Therefore, the increase in kT_reg_ cells with impaired mitochondria could be detrimental with aging. However, the function of kT_reg_ cells during aging and age-associated diseases requires further exploration.

Our findings demonstrate that, besides mitochondrial alterations, kT_reg_ cells harbor other signs of senescence. A recent study found that T_reg_ cells undergo senescence faster than conventional T cells through a ROS-dependent mechanism^64^. In addition, both mitochondrial decline and cellular senescence are tightly interconnected^31,32^. An important number of senescent-associated changes are dependent on mitochondrial function, especially the pro-inflammatory phenotype. Therefore, the pro-inflammatory phenotype observed in kT_reg_ cells could be a consequence of their mitochondrial dysfunction. The mitochondrial damage leads to the release of mitochondrial DNA (mtDNA) to the cytosol. mtDNA fragments trigger the activation of the cGAS–STING pathway, leading to the production of type I interferons, such as IFN-β, and pro-inflammatory cytokines, such as TNF or IL-6 (ref. ^65^), which are part of the SASP^66^. Cytosolic mtDNA, especially the oxidized mtDNA, also activates NLRP3 promoting the assembly of the inflammasome, a structure that leads to the activation of caspase I, therefore leading to the release of active IL-18 and IL-1β by the pyroptotic cell death^67^. As kT_reg_ cells harbor mitochondrial damage, they could also experience a release of mtDNA that would trigger their pro-inflammatory phenotype. Moreover, the mitochondrial dysfunction also contributes to lysosomal stress favoring a pro-inflammatory phenotype in CD4^+^ T cells^68^.

Surprisingly, there is recent evidence supporting the idea that senescence could be a reversible state. Chemotherapy-induced senescent cells can be released from senescence and reenter into the cell cycle^69^. In addition, tissue senescence can be transitorily induced to promote tissue regeneration in *Hydractinia symbiolongicarpus*^70^. Regarding CD4^+^ T cells, a recent study demonstrated that the expression of the senescence marker KLRG1 is transient in a subset of resident T_reg_ cells, despite their maintenance of an activated phenotype^71^. Because the specific ablation of mitochondria from senescent cells is sufficient to reverse many features of the senescent phenotype^72^, mitochondria are a promising target for interventions aimed at reducing the harmful effects of senescence in aging tissues. Interestingly, mitochondria dysfunction in exhausted CD8^+^ T cells can be reversed by scavenging ROS or boosting mitochondrial function with nicotinamide riboside^57,73,74^, suggesting that mitochondrial stress is reversible and that mitochondria could be key to restoring T cell function. Future research should clarify whether mitochondrial damage and other features of senescence are targetable in kT_reg_ cells.

Sustaining a proper function of T cells during aging is essential for healthy aging. Unraveling the changes in different T cell compartments will help to better understand the underlying mechanisms of age-associated immunological decline and, thus, to develop therapeutic interventions that enhance immunity in older adults.

## Methods

### Animal procedures

C57BL/6J HccRsd mice were purchased from Envigo or generated at the Centro de Biología Molecular Severo Ochoa (Madrid, Spain) animal facilities. All mice required for this study were bred and aged in specific-pathogen-free conditions in the animal facility of Centro de Biología Molecular Severo Ochoa (Madrid, Spain). All mice were housed in ventilated cages within animal rooms maintained under a 12 h–12 h light-dark cycle. Animal rooms were temperature and humidity controlled. Standard diet and water were available ad libitum. Both male and female mice were used in this study.

All the procedures with animals were previously evaluated and approved (PROEX 287/16 and PROEX 52.1/23) by the ethics commitee on animal experimentation of the CBMSO, the authorized commitee of the Spanish National Research Council or the Universidad Autónoma de Madrid, and the regional government (Comunidad de Madrid). All mice were checked for any macroscopic abnormalities (according to the Jackson guide ‘AGED C57BL/6J MICE FOR RESEARCH STUDIES’). Mice were used at different ages: young (less than 4 months of age), adult (4–20 months of age) and old (over 20 months of age).

CD3ε^−/−^ mice (JAX stock no. 004177) were kindly provided by Prof. B. Alarcón (CBM). CD45.1 (JAX stock no. 002014) mice were kindly provided by Dr. C. Cobaleda (CBM). Both strains were in C57BL/7 background.

#### IL-2 + IL-33 treatment

The IL-2 immune complex was prepared as previously described^43^. In brief, 1 µg of IL-2 (Peprotech, #212-12) was mixed with 5 µg of α-IL-2 (BioLegend, #503706) per mouse and incubated at room temperature (RT) for 30 min. Each mouse was intraperitoneally injected for three consecutive days. As a control, mice received only 200 μl of NaCl 0.9%. From the third day, IL-2 was combined with IL-33. 1 µg of IL-33 (BioLegend, #580506) was injected in 200 μl of NaCl per mouse for five consecutive days. As a control, mice received only 200 μl of NaCl.

#### Adoptive transfer of T_reg_ cells

For the study of the function of kT_reg_ cells in vivo, KLRG1^−^ and KLRG1^+^ T_reg_ cells (CD4^+^CD25^+^) were isolated by fluorescence-activated cell sorting (FACS) from IL-33-treated mice and injected into CD3ε^−/−^ young mice for three consecutive days. Approximately 8 × 10^5^ total T_reg_ cells were injected per mouse. Mice were weighed and monitored every 3 days for 4 months after the adoptive transfer and then were euthanized and the spleens were analyzed.

### Suppression assays

For the in vivo suppression assays, KLRG1^−^ and KLRG1^+^ T_reg_ cells (CD4^+^CD25^+^) were isolated by FACS from IL-33-treated mice. Naive CD4^+^ T cells were purified by magnetic isolation (Stemcell Technologies, #19765) from young CD45.1 mice. A total of 6 × 10^5^ naive CD4^+^ T cells alone or in combination with 1.5 × 10^5^ KLRG1^−^ or KLRG1^+^ T_reg_ cells were intravenously injected into CD3ε^−/−^ young mice. The animals were weighed and monitored every 3 days for 4 months after the adoptive transfer and then were euthanized and the colonic lamina propria was analyzed.

For the in vitro suppression assays, KLRG1^−^ and KLRG1^+^ T_reg_ cells (CD4^+^CD25^+^) were isolated by FACS from old mice. Naive CD4^+^ T cells were purified by magnetic isolation from young CD45.1 mice. CD45.1^+^ naive CD4^+^ T cells were labeled with 1 µM of CellTrace Violet (Invitrogen, C34571) in PBS for 30 min at 37 °C. A total of 2.5 × 10^4^ naive T_reg_ cells were incubated with 1.25 T_reg_ cells for 3 days. Cells were stimulated with Mouse T-Activator Dynabeads (Gibco, 11452D) following the manufacturer’s instructions. The percentage of suppression was determined as follows: 100 − (% of proliferating cells with T_reg_ cells)/(% of proliferating cells without T_reg_ cells).

### Human samples

Volunteer recruitment was performed through the GENYAL Clinical Trials Platform of IMDEA Alimentación (Madrid, Spain). This study was approved by the institutional Research Ethics Committee (IMDEA Food Foundation, IMD PI-052 and IMD PI-055) and performed in accordance with the principles of research involving human subjects stated in the Declaration of Helsinki (1964). All patients were clearly informed about the study methodology and provided written informed consent.

Peripheral blood samples were collected from a total of 117 volunteers belonging to two different population groups, whose inclusion criteria were as follows:

- Young healthy population (*n* = 42): 14 male and 28 female healthy volunteers aged between 18 and 25 years.
- Senior population (*n* = 75): 28 male and 47 female volunteers over 55 years of age.

Exclusion criteria were common for both study groups and included: decreased cognitive function, pregnancy or breastfeeding, severe chronic health conditions (for example, chronic kidney, liver and heart disease), immunodeficiencies and autoimmune diseases, and immunosuppressive or psychotropic pharmacological treatment. The results shown in the study apply to both sexes. The experimental groups have been defined by the age or the participants, and no sex- and gender-based analyses have been performed. Volunteers received financial compensation after the study.

### Tissue processing for flow cytometry

Mice were euthanized with CO_2_ followed by perfusion with cold PBS. The indicated tissues were extracted and processed as specified.

#### Spleen and lymph nodes

Lymph nodes were collected from inguinal, mesenteric, cervical and axillar areas. Spleen and lymph nodes were mashed and filtered through a 70-μm cell strainer. The cell suspension was centrifuged at 300*g* for 5 min at 4 °C. Red blood cells were removed using 5 ml of erythrocyte lysis buffer (ammonium chloride 0.15 M, sodium bicarbonate 0.01 M and EDTA 0.0001 M) for 5 min. Cells were washed, centrifuged, resuspended and counted.

#### Blood

Blood was extracted from either the facial vein or the heart in living or euthanized mice, respectively. The cell suspension was centrifuged at 300*g* for 5 min at 4 °C. Red blood cells were removed resuspending the cells in 5 ml of erythrocyte lysis buffer for 5 min. Cells were washed, centrifuged, resuspended and stained.

#### Colonic lamina propria

T cells from colonic lamina propria were isolated as previously reported^75^. Colon samples between the cecum and rectum were obtained and cleaned from fat and feces. Tissues were cut longitudinally, washed with cold PBS and then cut transversally into 1-cm-long fragments, mixed in prewarmed 5 mM EDTA, 14 mH HEPES, 10% FBS PBS and incubated under shaking at 180 rpm for 30 min at 37 °C. After washing with PBS, tissue pieces were then minced and mixed in prewarmed 25 mM HEPES, 10% FBS RPMI supplemented with 300 U ml^−1^ collagenase type VIII (Sigma, C2139) under shaking at 180 rpm for 45 min at 37 °C. The digested tissue was filtered through a 70-μm cell strainer, washed with 5 mM EDTA, 14 mH HEPES, 10% FBS PBS and centrifuged at 650*g* for 5 min at RT. To further enrich in leukocytes, supernatants were centrifuged in a 40%/70% Percoll gradient (Sigma, GE17-0891-01) at 750*g* for 20 min at RT with minimum acceleration and without brake. Isolated cells were washed with PBS and resuspended in 2% FBS RPMI for counting.

#### White adipose tissue

Gonadal white adipose tissue was obtained from the mouse abdominal cavity and mixed in 2 mg ml^−1^ BSA, 2% FBS RPMI supplemented with 2 mg ml^−1^ collagenase type II (Sigma, C6885) under shaking at 180 rpm for 40 min at 37 °C. The digested tissue was vertically rested to separate fat from the aqueous phases, which were obtained using an 18 G syringe. Then, the cell suspensions were filtered through a 70-μm cell strainer and washed with 2% FBS RPMI. Finally, erythrocytes were removed by incubation with a lysis buffer for 5 min at 4 °C, washed with 1 mM EDTA PBS and finally resuspended in 1 ml of 1 mM EDTA 2% FBS PBS for counting.

#### Peyer’s patches

Peyer’s patches were collected from the intestine and mashed into a 70-μm cell strainer. The cell suspension was centrifuged at 400*g* for 5 min at 4 °C. Finally, the cell pellets were resuspended in 1 ml of 2% FBS RPMI for counting.

#### Liver

Liver was collected and cut into prewarmed 25 mM HEPES, 10% FBS RPMI supplemented with 0.4 mg ml^−1^ collagenase type VIII (Sigma, C2139) under shaking at 180 rpm for 45 min at 37 °C. Digested tissue was filtered through a 70-μm cell strainer and centrifuged at 350*g* for 5 min at 4 °C. Red blood cells were removed using 5 ml of erythrocyte lysis buffer for 5 min. To further enrich in leukocytes, supernatants were centrifuged in a 40%/70% Percoll gradient (Sigma, GE17-0891-01) at 1,250*g* for 30 min at RT with acceleration on 6 and without brake. Isolated cells were washed with PBS and resuspended in ml 2% FBS RPMI for counting.

#### Bone marrow

Femurs and tibias were collected and the cells from the bone marrow were obtained by centrifuging the bones at 6,000*g* for 1 min. Red blood cells were removed using 5 ml of erythrocyte lysis buffer for 5 min. Cells were washed and resuspended in 2% FBS PBS for counting.

#### Human samples

Human blood samples were collected by venipuncture in an overnight fasting state. Three milliliters of blood were collected in TransFix/EDTA vacuum blood collection tubes (Cytomark) and preserved until the day of staining and cell acquisition.

### Flow cytometry

To differentiate between live and dead, the cells were first stained with the Zombie NIR Fixable Viability Kit (BioLegend, 423106, 1:3,000), the Zombie Yellow Fixable Viability Kit (BioLegend, 423104, 1:3,000) or the Ghost Dye Violet 540 (Tonbo Biosciences, 13-0879, 1:3,000) for 20 min at 4 °C. Then, the cells were washed with FACS staining buffer (PBS supplemented with 2% fetal bovine serum and 1 mM EDTA) and incubated with Fc receptor blocker purified rat anti-mouse anti-CD16/CD32 (BD Biosciences, 553142, 1:200) for 20 min at 4 °C. Cells were then incubated with primary antibodies for 20 min at 4 °C and were washed twice with FACS staining buffer. The following antibodies were diluted in Brilliant Stain Buffer (BD Biosciences, 566349) for surface antigen staining:

**Table:** 

Antigen	Fluorochrome	Dilution	Clone	Supplier	Catalog
CD38	Pacific Blue	1:500	90	BioLegend	102720
TIM3	BV480	1:200	5D12/TIM-3	BD Biosciences	747618
CD244.2 (2B4)	BV510	1:200	2B4	BD Biosciences	740115
CD44	BV570	1:200	IM7	BioLegend	103037
CD69	BV650	1:100	H1.2F3	BioLegend	104541
CD62L	BV711	1:400	MEL-14	BioLegend	104445
CD95	BV750	1:100	Jo2	BD Biosciences	747413
KLRG1	BV785	1:200	2F1	BioLegend	138429
CD223 (LAG3)	BB515	1:200	C9B7W	BD Biosciences	566210
CD49d	PerCP-Cy5.5	1:100	R1-2	BioLegend	103619
PD-1	PerCP-eFluor710	1:200	J43	ThermoFisher	46-9985-80
ST2	PerCP-eFluor710	1:200	RMST2-33	ThermoFisher	46-9333-82
NKG2A	PE	1:200	16A11	BioLegend	142804
NKG2D	PE-Dazzle594	1:200	CX5	BioLegend	130214
CD25	PE Cy5	1:400	PC61	BioLegend	102007
CD8	PE-Fire700	1:1000	53-6.7	BioLegend	100792
CD28	APC	1:50	E18	BioLegend	122016
CD153	R718	1:200	RM153	BD Biosciences	751871
CD27	APC-Cy7	1:400	LG.3A10	BioLegend	124226
CD4	APC-Fire810	1:1000	Gk1.5	BioLegend	100480

For intracellular staining, after staining for membrane markers, the cells were fixed and permeabilized using the FOXP3/Transcription Factor Staining Kit (eBioscience, 00-5523-00) for 20 min at RT and darkness. To assess cytokine production, cells were stimulated for 4 h with 50 ng ml^−1^ phorbol 12-myristate 13-acetate (PMA) (ThermoFisher, 356150010) and 1 μg ml^−1^ ionomycin (Tocris, 1704) in the presence of brefeldin A (eBiosciences, 00-4506-51). Cells were then stained the following intracellular antibodies:

**Table:** 

Antibody	Fluorochrome	Dilution	Clone	Supplier	Catalog
IL-10	Pacific Blue	1:100	JES5-16E3	BioLegend	505020
IL-17	PE	1:100	TC11-18H10.1	BioLegend	506904
TNF	APC	1:100	MP6-XT22	BioLegend	506308
IFN-γ	Spark NIR 685	1:100	XMG1.2	BioLegend	505861
MAF	eFluor450	1:100	sym0F1	ThermoFisher	48-9855-41
FOXP3	FITC	1:200	FJK-165	ThermoFisher	11-5773-82
RORγt	PE	1:100	Q31-378	BD Biosciences	562607
T-BET	AlexaFluor594	1:100	4B10	BioLegend	644834
T-BET	APC	1:100	4B10	BioLegend	17-5825-82
TOX	eFluor660	1:200	TXRX10	ThermoFisher	50-6502-80
γH2AX	–	1:300	20E3	CellSignaling	9718
P16	–	1:200	Polyclonal	ThermoFisher	PA5-119712
P21	Alexa Fluor 647	1:400	Polyclonal	Abcam	ab237265
Donkey anti-rabbit	Alexa Fluor 647	1:500	Polyclonal	ThermoFisher	A-31573

All flow cytometry experiments from mouse samples were performed with four-laser (violet, blue, yellow-green and red) or five-laser Aurora analyzers (Cytek Biosciences). Data were analyzed with the FlowJo v10.5.3 software (BD Biosciences). Gating strategies were set on the basis of fluorescence minus one controls, unstained samples and unstimulated samples (when needed). All the samples in the experiment excluded dead cells, clumps and debris.

In human samples, each tube contained at least 2 × 10^6^ human whole blood cells. Cells were labeled by incubation with appropriate fluorescence-conjugated antibodies for 15 min at RT in the dark. Cells were then lysed with 2 ml of FACS lysing solution (BD Biosciences) for 10 min and centrifuged at 500*g* for 5 min at RT. Then, the cells were washed with 5 ml of PBS. The following antibodies were used for surface antigen staining:

**Table:** 

Antibody	Fluorochrome	Dilution	Clone	Supplier	Catalog
CD8	BUV496	1:100	RPA-T8	BD Biosciences	612942
CD25	BUV615	1:100	2A3	BD Biosciences	612996
CD19	BUV661	1:100	1D3	BD Biosciences	612971
CD45	BV510	1:100	HI30	BD Biosciences	563204
CD4	BV786	1:100	SK3	BD Biosciences	664528
CD127	PE	1:100	HIL-7R-M21	BD Biosciences	561028
CD3	APC	1:100	HIT3a	BD Biosciences	555342
KLRG1	APC-Cy7	1:100	2F1/KLRG1	BioLegend	138426

Experiments with human samples were performed in a BD FACSymphony A5 SORP flow cytometer (BD Biosciences). To generate comparable results among patients and over time, the photomultiplier voltages were adjusted to unlabeled lysed whole blood cells to obtain optimal photomultiplier tube voltages for the resolution of dim cell populations. The target values resulting of the optimization were used for subsequent calibrations to maintain instrument standardization. When possible, at least 40,000 events of CD4 population were acquired to reach the maximum T_reg_ events. Data were analyzed using the FlowJoTM v.10 software.

The gating strategy for identifying kT_reg_ cells is illustrated in the Extended Data Fig. 6. In brief, first, doublets and debris were excluded in forward scatter (FSC) – side scatter (SSC) dot plots. Then, a region in CD45 and SSC parameters were used to discriminate leukocytes. Next, lymphocytes were gated in a SSC – CD4 dot plot. Lymphoid cells were further cleaned in a SSC – CD45 plot. After that, CD3^+^CD19^−^ T cells were gated and, among them, CD4^+^ T lymphocytes were distinguished. The T_reg_ cells were identified, within the CD4^+^ T cells, by gating the CD25^hi^CD27^lo^ population. Then, the expression of KLRG1 was analyzed in T_reg_ cells. To have a negative control to properly gate KLRG1^+^ cells, the gate was established in B cells.

#### Analysis of mitochondrial fitness

Analysis of mitochondrial mass and MMP was performed by flow cytometry in cells labeled for 30 min with 50 nM MitoTracker Green FM (Invitrogen, M7514), 200 nM MitoTracker DeepRed FM (Invitrogen, M22426), 40 nM Image-iT TMRM (Invitrogen, I34361) or 50 nM MitoTracker Red CMXRos (Invitrogen, M7512) in RPMI medium with no FBS in a 37 °C and 5% CO_2_ incubator before extracellular staining. After incubation, cells were washed with FACS staining buffer and incubated with antibodies for extracellular staining.

### Dimensional reduction and clustering analysis of flow cytometry data

Dimensional reduction and clustering analysis of flow cytometry data was done using OMIQ (Dotmatics). First, nonlymphocyte cells, doublets and dead cells were excluded on the basis of viability staining and FSC and SSC parameters. Then, 15,000 CD4^+^ cells from each sample were subsampled for further analysis. For dimensional reduction, the Uniform Manifold Approximation and Projection (UMAP) algorithm was applied with the following parameters: neighbors = 15, minimum distance = 0.4, components = 2, learning rate = 1, epochs = 200. For unbiased clustering, the Cluster-X algorithm was applied with alpha = 0.001. Mean fluorescence intensity for each marker projected on the UMAP plots was used to infer the cluster’s identity, and similar clusters were combined.

### RNA-seq

rT_reg_, aT_reg_ and kT_reg_ cells were isolated from young and old mice by FACS. RNA was extracted with the RNeasy Micro Kit (Qiagen, 74004) following manufacturer’s instructions. Sample quality was measured using the Qubit 3.0 fluorimeter for sample concentration and Agilent 5400 for fragment analysis. SMART-Seq V4 Ultra Low Input RNA kit for Sequencing 480 Rxns (Takarabio, 634893) was used for efficient cDNA synthesis and library preparation. After amplification, cDNA was purified using AMPure XP beads (Beckman Coulter, A63882) to remove any contaminants and ensure high-quality cDNA. The library was checked with Qubit and real-time PCR for quantification and bioanalyzer for size distribution detection. Quantified libraries were pooled and sequenced on Illumina Illumina Novaseq X plus using a PE150 (pair-end 150 base pair) strategy to produce 6G of data.Fastq files quality check was performed using FastQC v0.11.9. RNA-seq reads were mapped to the *Mus musculus* reference genome, GRCm39, using Hisat2 v2.2.1 software. Reads were then preprocessed with SAMtools v1.13 to transform Sequence Alignment/Map files into Binary Alignment/Map files and sorted. The number of reads covered by each gene is calculated by HTSeq-Count v1.99.2. Downstream data analysis was performed with R v4.4.1. Analysis of differentially expressed genes was performed using DESeq2 v1.44.0. Genes with *P* < 0.05 and |log_2_fold change| >log_2_(1.5) were determined to show statistically significant differences in group comparison. Over-representation analysis and gene set enrichment analysis were performed using clusterProfiler v4.12.0 package in GO, KEGG, WikiPathways, Reactome and the Hallmarks of the Molecular signatures database. Principal component analysis (PCA) plots and volcano plot were visualized using ggplot2 v3.5.1. Heat maps were visualized using heatmap v1.0.12.

### Analysis of cytokine secretion by T_reg_ subsets using Multiplex

rT_reg_, aT_reg_ or kT_reg_ cells were isolated from IL-33-treated young mice by FACS. A total of 5.5 × 10^5^ cells were incubated in 200 µl of complete RPMI medium supplemented with 10% FBS in resting conditions or in presence of Mouse T-Activator Dynabeads for 24 h. The cells were centrifuged at 300*g* for 5 min, and the supernatant was used for cytokine detection with magnetic bead technology (Invitrogen, Cytokine & Chemokine 26-Plex Mouse ProcartaPlex Panel 1).

### Electron microscopy

For electron microscopy, splenocytes from old mice were labeled for 30 min with 50 nM MitoTracker Green FM and 200 nM Mitotracker DeepRed FM before extracellular staining. Cells were then labeled with a fluorochrome-conjugated antibody against CD4, and MtG^hi^MtDR^hi^ and MtG^lo^MtDR^lo^ alive CD4^+^ T cells were isolated by FACS. After sorting, cells were fixed with 4% paraformaldehyde, 2% glutaraldehyde diluted in 0.1 M phosphate buffer. Cells were postfixed with 1% osmiun tetroxide and 1% potassium ferrocyanide for 60 min. After washing, incubation with 0.15% tannic acid in buffer phosphate 0.1 M for 1 min was achieved. After washing, cells were counterstained with uranyl acetate 2% for 1 h. Then, cells were dehydrated with lowering concentrations of ethanol and embedded in resin EPON. The preparations were examined with a transmission electron microscope (JEM1400 Flash, Jeol), and images were acquired with a CMOS Oneview camera (Gatan).

### Reanalysis of scRNA-seq data

scRNA-seq data were extracted from a previously published work^5^ and reanalyzed with the Seurat package v4.2.0 in R v4.1.3. Variable genes were identified with the FindVariablesFeatures function across the range of expression values and used to perform a PCA with the RunPCA function. Clustering was performed with the FindClusters function with the Leiden algorithm and the first 20 principal components. Cluster identification was done with the FindMarkers function in each subset with a minimum log fold change of 0.25 and a *P* value <10^-3^. The kT_reg_ cluster was separated by using the FindSubCluster function in the aT_reg_ cluster and then identified with the FindMarkers function.

### Statistics and reproducibility analysis

Unless otherwise specified, *n* represents the number of individual biological replicates and is represented in graphs as one dot per sample. All the data are extracted from one representative experiment (*n* ≥ 3 per group) of a minimum of two separate experiments. Flow cytometry plots are representative of at least three replicates. No statistical method was used to predetermine sample size, but a minimum of three samples were used per experimental group and condition. Data collection and analysis were not performed blind to the age of the mice.

For statistical analysis, GraphPad Prism (version 9) was used. Data are expressed as mean ± s.e.m., unless otherwise indicated. Outliers were identified by the ROUT method (5%) and removed. Comparisons for two groups were calculated using unpaired two-tailed Student’s *t*-tests (for two groups meeting the normal distribution criteria) or Mann–Whitney *U* test (for two groups without normal distribution) according to the Shapiro–Wilk normality test. When comparing different populations within the same mouse, comparisons were calculated using two-tailed paired Student’s *t*-tests (for two groups meeting the normal distribution criteria) or Wilcoxon test (for two groups without normal distribution) according to the Shapiro–Wilk normality test. Comparisons for more than two groups were calculated using one-way analysis of variance (ANOVA) with Tukey’s correction for multiple comparisons (for three or more groups meeting the normal distribution criteria) or Kruskal–Wallis test with Dunn’s correction for multiple comparisons (for three or more groups without normal distribution) according to the Shapiro–Wilk normality test. When comparing at least three distinct populations within the same mouse, comparisons were calculated using repeated measures (RM) one-way ANOVA with Tukey’s correction for multiple comparisons (for samples meeting the normal distribution criteria) or Friedman test with Dunn’s correction for multiple comparisons (for at least three groups without normal distribution) according to the Shapiro–Wilk normality test.

The statistical significance is represented as follows: *P < 0.05, **0.05 < *P* < 0.01; ***0.01 < *P* < 0.001, ****P < 0.001.

### Reporting summary

Further information on research design is available in the Nature Portfolio Reporting Summary linked to this article.

## Supplementary information

Reporting Summary

Supplementary Table 1 Differentially expressed genes in the kT_reg_ cluster.

## Source data

Source Data Fig. 1 Statistical source data.

Source Data Fig. 2 Statistical source data.

Source Data Fig. 3 Statistical source data.

Source Data Fig. 4 Statistical source data.

Source Data Fig. 5 Statistical source data.

Source Data Fig. 6 Statistical source data.

Source Data Fig. 7 Statistical source data.

Source Data Fig. 8 Statistical source data.

Source Data Extended Data Fig. 1 Statistical source data.

Source Data Extended Data Fig. 2 Statistical source data.

Source Data Extended Data Fig. 3 Statistical source data.

Source Data Extended Data Fig. 4 Statistical source data.

Source Data Extended Data Fig. 5 Statistical source data.

## Data Availability

All data supporting the findings of this study are available within the Article and its Supplementary Information. The scRNA-seq dataset reanalyzed is publicly available (Single Cell Portal; accession number SCP490)^5^. The RNA-seq dataset generated during the current study is available in the Gene Expression Omnibus repository (GSE279926). The rest of the data were originally generated.
