# Supplementary material for: KLRG1 identifies regulatory T cells with mitochondrial alterations that accumulate with aging
Source: Nat Aging. 2025 Apr 30;5(5):799–815. doi: 10.1038/s43587-025-00855-9 (PMC12092250; doi:10.1038/s43587-025-00855-9)
Supplement: Supplementary file 1 — Reporting Summary [file 43587_2025_855_MOESM1_ESM.pdf]

nature portfolio

Corresponding author(s): María Mittelbrunn

Last updated by author(s): Feb 9, 2025

Reporting Summary

Nature Portfolio wishes to improve the reproducibility of the work that we publish. This form provides structure for consistency and transparency in reporting. For further information on Nature Portfolio policies, see our Editorial Policies and the Editorial Policy Checklist.

Statistics

For all statistical analyses, confirm that the following items are present in the figure legend, table legend, main text, or Methods section.

n/a	Confirmed	
☐	☒	The exact sample size (*n*) for each experimental group/condition, given as a discrete number and unit of measurement
☐	☒	A statement on whether measurements were taken from distinct samples or whether the same sample was measured repeatedly
☐	☒	The statistical test(s) used AND whether they are one- or two-sided<br>*Only common tests should be described solely by name; describe more complex techniques in the Methods section.*
☐	☒	A description of all covariates tested
☐	☒	A description of any assumptions or corrections, such as tests of normality and adjustment for multiple comparisons
☐	☒	A full description of the statistical parameters including central tendency (e.g. means) or other basic estimates (e.g. regression coefficient) AND variation (e.g. standard deviation) or associated estimates of uncertainty (e.g. confidence intervals)
☐	☒	For null hypothesis testing, the test statistic (e.g. F, t, r) with confidence intervals, effect sizes, degrees of freedom and P value noted<br>*Give P values as exact values whenever suitable.*
☒	☐	For Bayesian analysis, information on the choice of priors and Markov chain Monte Carlo settings
☒	☐	For hierarchical and complex designs, identification of the appropriate level for tests and full reporting of outcomes
☒	☐	Estimates of effect sizes (e.g. Cohen's *d*, Pearson's *r*), indicating how they were calculated

Our web collection on statistics for biologists contains articles on many of the points above.

Software and code

Policy information about availability of computer code

Data collection	Flow cytometry data were collected with commercial softwares. SpectroFlo was used for acquisition of mouse samples in Aurora flow cytometers (Cytek Biosciences). FACS Diva was used for acquisition of human samples data in a BD FACSymphony A5 SORP flow cytometer (BD Biosciences)
Data analysis	Flow Cytometry<br>Flow cytometry data were analyzed using the FlowJoTM v.10 software (BD Biosciences).<br>For multiparametric analysis, dimensional reduction and clustering analysis of flow cytometry data was done using OMIQ (Dotmatics).<br><br>Electron microscopy<br>Images were visualized and quantified with FIJI software (NIH).<br><br>scRNAseq<br>scRNAseq data was extracted from a previously published work 5 and reanalyzed with the Seurat package v4.2.0 in R v4.1.3. Variable genes were identified with the FindVariablesFeatures function across the range of expression values and used to perform a principal component analyses (PCA) with the RunPCA function. Clustering was performed with the FindClusters function with the Leiden algorithm and the first 20 principal components. Clusters identification was done with the FindMarkers function in each subset with a minimum log fold change of 0.25 and a P-value <10-3. kTreg cluster was separated by using FindSubCluster function in the aTreg cluster and then identified with the FindMarkers function.<br><br>RNAseq<br>Fastq files quality check was performed using FastQC v0.11.9. RNA-sequencing reads were mapped to the Mus musculus reference genome,

GRCm39, using Hisat2 v2.2.1 software. Reads were then preprocessed with SAMtools v1.13 to transform Sequence Alignment/Map files into Binary Alignment/Map files and sorted. The number of reads covered by each gene is calculated by HTSeq-Count v1.99.2. Downstream data analysis was performed with R v4.4.1. DEG analysis was performed using DESeq2 v1.44.0. Genes with p < 0.05 and |log2FC| > log2(1.5) were determined to show statistically significant differences in group comparison. Over-Representation Analysis (ORA) and Gene Set Enrichment Analysis (GSEA) were performed using clusterProfiler v4.12.0 package in GO, KEGG, WikiPathways, Reactome and the Hallmarks of the Molecular signatures database. PCA plots, Volcano plot and heatmaps were visualized by using ggplot2 v3.5.1. and heatmap v1.0.12, respectively.

Statistic analysis
Data anlaysis and representation was performed with GraphPad Prism v9.1.1 (Dotmatics).

For manuscripts utilizing custom algorithms or software that are central to the research but not yet described in published literature, software must be made available to editors and reviewers. We strongly encourage code deposition in a community repository (e.g. GitHub). See the Nature Portfolio guidelines for submitting code & software for further information.

Data

Policy information about availability of data

All manuscripts must include a data availability statement. This statement should provide the following information, where applicable:

- Accession codes, unique identifiers, or web links for publicly available datasets
- A description of any restrictions on data availability
- For clinical datasets or third party data, please ensure that the statement adheres to our policy

All data supporting the findings of this study are available within the paper and its Supplementary Information.

The scRNAseq data used in Extended Figure 2 and Extended Figure 3 were collected from "Aging promotes reorganization of the CD4 T cell landscape toward extreme regulatory and effector phenotypes" Elyahu et al. 2019, Science Advances (doi: 10.1126/sciadv.aaw8330).

RNAseq data is available and can be found under GEO accession number GSE279926.

The rest of the data were originally generated.

Research involving human participants, their data, or biological material

Policy information about studies with human participants or human data. See also policy information about sex, gender (identity/presentation), and sexual orientation and race, ethnicity and racism.

Reporting on sex and gender	Consent was obtained to communicate and share dissociated data at the individual level.<br>Information regarding the sex of the participants was collected. Sex was determined based on self-reporting by the volunteers.<br>No information regarding the gender of the participants was collected.<br>A total of 14 males and 28 females participated in the study within the young population subgroup, and a total of 28 males and 47 females within the senior population subgroup.<br>The results shown in the study apply to both sexes. The experimental groups have been defined by the age or the participants and no sex- and gender-based analyses have been performed.
Reporting on race, ethnicity, or other socially relevant groupings	Information on ethnicity was self-reported by study participants. All volunteers (100%) in the study self-reported their ethnicity as European Mediterranean.<br>This information was collected with the sole purpose of understanding the possible genetic variability of the population, local adaptations and cultural influences. In this research no categorization by race, ethnicity, or other socially relevant groupings was performed.
Population characteristics	In accordance with the objectives of the study, volunteers were recruited according to their age. Peripheral blood samples were collected from a total of 144 volunteers belonging to two different population groups, whose inclusion criteria were:<br>- Young healthy population (n=78): Healthy volunteers aged between 18 and 25 years.<br>- Senior population (n=66): Volunteers over 55 years of age.<br>Exclusion criteria were common for both study groups and included: decreased cognitive function, pregnancy or breastfeeding, severe chronic health conditions (chronic kidney/liver/heart disease...), immunodeficiencies and autoimmune diseases, and immunosuppressive or psychotropic pharmacological treatment.
Recruitment	Volunteers recruitment was performed through the GENYAL Clinical Trials Platform of IMDEA Alimentación (Madrid, Spain), , which has a database of more than 2,000 active volunteers, as well as through the dissemination of recruitment posters on social networks of the IMDEA Institute. All patients were clearly informed about the study methodology and provided written informed consent.
Ethics oversight	All procedures involving human participants in this research were conducted in accordance with the Declaration of Helsinki. Informed consent was obtained from all human research participants. The Ethics committee approving this research was the Research Ethics Committee of the IMDEA Food Foundation. The approval codes for both clinical research protocols were IMD code: PI-052 (young population) and IMD code: PI-052 (senior population).

Note that full information on the approval of the study protocol must also be provided in the manuscript.

Field-specific reporting

Please select the one below that is the best fit for your research. If you are not sure, read the appropriate sections before making your selection.

☒ Life sciences ☐ Behavioural & social sciences ☐ Ecological, evolutionary & environmental sciences

For a reference copy of the document with all sections, see nature.com/documents/nr-reporting-summary-flat.pdf

Life sciences study design

All studies must disclose on these points even when the disclosure is negative.

Sample size

In all the experiments of this study, no statistical method was used to predetermine sample size, but a minimum of four samples were used per experimental group and condition. Four individual samples were considered as the minimun to have enough information for statistical tests and still reduce the number of animals used. In addition, most of the experiments were performed with 5 or 6 mice because they were initially caged together.

Data exclusions

Mice with signs of unhealthy aging (dermatitis, tumours) were removed before performing the experiments.
When the experimental groups had more than 4 samples, outliers were identified by the ROUT method (5%) and removed. Since many analytical techniques rely on measures of central tendency like mean, outliers with extreme values can unduly influence calculations. The exclusion criteria were pre-established and applied to all the experiments performed.

Replication

Representative plots and graphs summarize results of at least two independent experiments. All attempts at replication were successful.

Randomization

Mice were caged together and grouped by their date of birth for this study.
Human samples were grouped solely based on age.

Blinding

In this study, experiments and data collection were primarly conducted in a no-blinded manner because the appearence of the animal is a strong indivative of its age and therefore its experimental group.
Human samples were blinded for their analysis. Human samples were anonymized during collection and analyzed. Deanonymization and assignment to experimental groups were performed after analysis.

Reporting for specific materials, systems and methods

We require information from authors about some types of materials, experimental systems and methods used in many studies. Here, indicate whether each material, system or method listed is relevant to your study. If you are not sure if a list item applies to your research, read the appropriate section before selecting a response.

Materials & experimental systems

n/a	Involved in the study	
☐	☒	Antibodies
☒	☐	Eukaryotic cell lines
☒	☐	Palaeontology and archaeology
☐	☒	Animals and other organisms
☒	☐	Clinical data
☒	☐	Dual use research of concern
☒	☐	Plants

Methods

n/a	Involved in the study	
☒	☐	ChIP-seq
☐	☒	Flow cytometry
☒	☐	MRI-based neuroimaging

Antibodies

Antibodies used

Antibodies for mouse samples:

Antibody	Fluorochrome	Clone	Supplier	Dilution	Catalog
1. CD38	Pacific Blue	90	Biolegend	1:500	102720
2. TIM3	BV480	5D12/TIM-3	BD Biosciences	1:200	747618
3. CD244.2 (2B4)	BV510	2B4	BD Biosciences	1:200	740115
4. CD44	BV570	IM7	Biolegend	1:200	103037
5. CD69	BV650	H1.2F3	Biolegend	1:100	104541
6. CD62L	BV711	MEL-14	Biolegend	1:400	104445
7. CD95	BV750	Jo2	BD Biosciences	1:100	747413
8. KLRG1	BV785	2F1	Biolegend	1:200	138429
9. CD223 (LAG3)	BB515	C9B7W	BD Biosciences	1:200	566210
10. CD49d	PerCP Cy5.5	R1-2	Biolegend	1:100	103619
11. PD1	PerCP eFluor710	J43	Thermofisher	1:200	46-9985-80
12. ST2	PerCP eFluor710	RMST2-33	Thermofisher	1:200	46-9333-82
13. NKG2A	PE	16A11	Biolegend	1:200	142804
14. NKG2D	PE-Dazzle594	CX5	Biolegend	1:200	130214

15. CD25	PE Cy5	PC61	Biolegend	1:400	102007
16. CD8	PE Fire700	53-6.7	Biolegend	1:1000	100792
17. CD28	APC	E18	Biolegend	1:50	122016
18. CD153	R718	RM153	BD Biosciences	1:200	751871
19. CD27	APC Cy7	LG.3A10	Biolegend	1:400	124226
20. CD4	APC Fire810	Gk1.5	Biolegend	1:1000	100480
21. IL-10	Pacific Blue	JES5-16E3	Biolegend	1:100	505020
22. IL-17A	PE	TC11-18H10.1	Biolegend	1:100	506904
23. TNF	APC	MP6-XT22	Biolegend	1:100	506308
24. IFN-γ	Spark NIR 685	XMG1.2	Biolegend	1:100	505861
25. MAF	eFluor450	sym0F1	ThermoFisher	1:100	48-9855-41
26. FOXP3	FITC	FJK-165	ThermoFisher	1:200	11-5773-82
27. RORγt	PE	Q31-378	BD Biosciences	1:100	562607
28. T-BET	AlexaFluor594	4B10	Biolegend	1:100	644834
29. T-BET	APC	4B10	ThermoFisher	1:100	17-5825-82
30. TOX	eFluor660	TXRX10	ThermoFisher	1:200	50-6502-80
31. γH2AX		20E3	CellSignaling	1:300	9718
32. P16	-	Polyclonal	ThermoFisher	1:200	PA5-119712
33. P21	Alexa Fluor 647	Polyclonal	Abcam	1:400	ab237265
34. Donkey a-Rabbit	Alexa Fluor 647	Polyclonal	ThermoFisher	1:500	A-31573

Antibodies for human samples:

Antibody	Fluorochrome	Clone	Supplier	Dilution	Catalog
1. CD8	BUV496	RPA-T8	BD Biosciences	1:100	612942
2. CD25	BUV615	2A3	BD Biosciences	1:100	612996
3. CD19	BUV661	1D3	BD Biosciences	1:100	612971
4. CD45	BV510	HI30	BD Biosciences	1:100	563204
5. CD4	BV786	SK3	BD Biosciences	1:100	664528
6. CD127	PE	HIL-7R-M21	BD Biosciences	1:100	561028
7. CD3	APC	HIT3a	BD Biosciences	1:100	555342
8. KLRG1	APC Cy7	2F1/KLRG1	Biolegend	1:100	138426

Validation

All the antibodies used for flow cytometry are comercially available. they are extensively validated in the literature, and validation data are available on manufacturers's websites:

Antibodies for mouse samples:
1. https://www.biolegend.com/en-us/products/pacific-blue-anti-mouse-cd38-antibody-6652
2. https://www.bdbiosciences.com/en-us/products/reagents/flow-cytometry-reagents/research-reagents/single-color-antibodies-ruo/bv480-mouse-anti-mouse-cd366-tim-3.747618?tab=product_details
3. https://www.bdbiosciences.com/en-us/products/reagents/flow-cytometry-reagents/research-reagents/single-color-antibodies-ruo/bv510-mouse-anti-mouse-cd244-2.740115?tab=product_details
4. https://www.biolegend.com/en-us/products/brilliant-violet-570-anti-mouse-human-cd44-antibody-7386
5. https://www.biolegend.com/en-us/products/brilliant-violet-650-anti-mouse-cd69-antibody-13310
6. https://www.biolegend.com/en-us/products/brilliant-violet-711-anti-mouse-cd62l-antibody-10317
7. https://www.bdbiosciences.com/en-us/products/reagents/flow-cytometry-reagents/research-reagents/single-color-antibodies-ruo/bv750-hamster-anti-mouse-cd95-fas.747413?tab=product_details
8. https://www.biolegend.com/en-us/products/brilliant-violet-785-anti-mouse-human-klrg1-mafa-antibody-13682
9. https://www.bdbiosciences.com/en-us/products/reagents/flow-cytometry-reagents/research-reagents/single-color-antibodies-ruo/bb515-rat-anti-mouse-cd223.566210?tab=product_details
10. https://www.biolegend.com/en-us/products/percp-cyanine5-5-anti-mouse-cd49d-antibody-9901
11. https://www.thermofisher.com/antibody/product/CD279-PD-1-Antibody-clone-J43-Monoclonal/46-9985-80
12. https://www.thermofisher.com/antibody/product/IL-33R-ST2-Antibody-clone-RMST2-33-Monoclonal/46-9333-82
13. https://www.biolegend.com/en-us/products/pe-anti-mouse-cd159a-nkg2ab6-antibody-7543
14. https://www.biolegend.com/en-us/products/pedazzle-594-anti-mouse-cd314-antibody-15542
15. https://www.biolegend.com/en-us/products/pe-anti-mouse-cd25-antibody-424
16. https://www.biolegend.com/en-us/products/pefire-700-anti-mouse-cd8a-antibody-19782
17. https://www.biolegend.com/en-us/products/apc-anti-mouse-cd28-antibody-3781
18. https://www.bdbiosciences.com/en-us/products/reagents/flow-cytometry-reagents/research-reagents/single-color-antibodies-ruo/r718-rat-anti-mouse-cd153.751871?tab=product_details
19. https://www.biolegend.com/en-us/products/apc-cyanine7-anti-mouse-rat-human-cd27-antibody-11905
20. https://www.biolegend.com/en-us/products/apc-fire-810-anti-mouse-cd4-antibody-19552
21. https://www.biolegend.com/en-us/products/pacific-blue-anti-mouse-il-10-antibody-6145
22. https://www.biolegend.com/en-us/products/pe-anti-mouse-il-17a-antibody-1633
23. https://www.biolegend.com/en-us/products/apc-anti-mouse-tnf-alpha-antibody-975
24. https://www.biolegend.com/en-us/products/spark-nir-685-anti-mouse-ifn-gamma-antibody-21051
25. https://www.thermofisher.com/antibody/product/c-MAF-Antibody-clone-sym0F1-Monoclonal/48-9855-42
26. https://www.thermofisher.com/antibody/product/FOXP3-Antibody-clone-FJK-16s-Monoclonal/11-5773-82
27. https://www.bdbiosciences.com/en-us/products/reagents/flow-cytometry-reagents/research-reagents/single-color-antibodies-ruo/pe-mouse-anti-mouse-ror-t.562607?tab=product_details
28. https://www.biolegend.com/en-us/products/alexa-fluor-594-anti-t-bet-antibody-15452
29. https://www.thermofisher.com/antibody/product/T-bet-Antibody-clone-eBio4B10-4B10-Monoclonal/17-5825-82
30. https://www.thermofisher.com/antibody/product/TOX-Antibody-clone-TXRX10-Monoclonal/50-6502-80
31. https://www.cellsignal.com/products/primary-antibodies/phospho-histone-h2a-x-ser139-20e3-rabbit-mab/9718
32. https://www.thermofisher.com/antibody/product/p16INK4a-Antibody-Polyclonal/PA5-119712
33. https://www.abcam.com/en-us/products/primary-antibodies/alexa-fluor-647-p21-antibody-epr18021-ab237265?srsltid=AfmBOoqEnFws1c4Ge-gnZi_JoPu0MQXzrbnQu3EJMh9YuZMV0fVOZ2Ds

34. https://www.thermofisher.com/antibody/product/Donkey-anti-Rabbit-IgG-H-L-Highly-Cross-Adsorbed-Secondary-Antibody-Polyclonal/A-31573

Antibodies for human samples:
1. https://www.bdbiosciences.com/en-us/products/reagents/flow-cytometry-reagents/research-reagents/single-color-antibodies-ruo/buv496-mouse-anti-human-cd8.612942?tab=product_details
2. https://www.bdbiosciences.com/en-us/products/reagents/flow-cytometry-reagents/research-reagents/single-color-antibodies-ruo/buv615-mouse-anti-human-cd25.612997?tab=product_details
3. https://www.bdbiosciences.com/en-us/products/reagents/flow-cytometry-reagents/research-reagents/single-color-antibodies-ruo/buv661-rat-anti-mouse-cd19.612971?tab=product_details
4. https://www.bdbiosciences.com/en-us/products/reagents/flow-cytometry-reagents/research-reagents/single-color-antibodies-ruo/bv510-mouse-anti-human-cd45.563204?tab=product_details
5. https://www.bdbiosciences.com/en-us/products/reagents/flow-cytometry-reagents/clinical-diagnostics/single-color-antibodies-asr-ivd-ce-ivd/cd4-sk3-bv786.664528?tab=product_details
6. https://www.bdbiosciences.com/en-us/products/reagents/flow-cytometry-reagents/research-reagents/single-color-antibodies-ruo/pe-mouse-anti-human-cd127.557938?tab=product_details
7. https://www.bdbiosciences.com/en-us/products/reagents/flow-cytometry-reagents/research-reagents/single-color-antibodies-ruo/apc-mouse-anti-human-cd3.555342?tab=product_details
8. https://www.biolegend.com/en-us/products/apc-cyanine7-anti-mouse-human-klrg1-mafa-antibody-12486

Animals and other research organisms

Policy information about studies involving animals; ARRIVE guidelines recommended for reporting animal research, and Sex and Gender in Research

Laboratory animals

C57BL/6J HccRsd mice were used in this study. Mice were prurchased from Envigo or generated at the Centro de Biología Molecular Severo Ochoa (Madrid, Spain) animal facilities.
CD3e-/- mice (JAX stock #004177) and CD45.1 mice (JAX stock #002014) were generated at the Centro de Biología Molecular Severo Ochoa (Madrid, Spain) animal facilities.
All mice required for this study were bred and aged in specific-pathogen-free conditions in the animal facility of Centro de Biología Molecular Severo Ochoa (Madrid, Spain). All mice were housed in ventilated cages within animal rooms maintained under a 12-12 light-dark cycle. Animal rooms were temperature and humidity controlled. Standard diet and water were available ad libitum.
Ages of the mice are indicated in each figure.

Wild animals

The study did not involve wild animals.

Reporting on sex

Findings apply to both sexes of mice. Both male and female were used indiscriminately in the experiments. However, most of the experiments were performed with females due to the possibility to recage them.

Field-collected samples

The study did not involve field-collected samples.

Ethics oversight

All the procedures with animals were previously evaluated and approved (PROEX 287/16 and PROEX 52.1/23) by the Ethics commitee on animal experimentation of the CBMSO, the authorized commitee of the Spanish National Research Council or the Universidad Autónoma de Madrid and the regional government (Comunidad de Madrid). All mice were checked for any macroscopic abnormalities (according to the Jackson guide “AGED C57BL/6J MICE FOR RESEARCH STUDIES”). Mice were used at different ages: young (less than 4 months of age), adult (4 to 20 months of age) and old (over 20 months of age).

Note that full information on the approval of the study protocol must also be provided in the manuscript.

Flow Cytometry

Plots

Confirm that:

☒ The axis labels state the marker and fluorochrome used (e.g. CD4-FITC).

☒ The axis scales are clearly visible. Include numbers along axes only for bottom left plot of group (a 'group' is an analysis of identical markers).

☒ All plots are contour plots with outliers or pseudocolor plots.

☒ A numerical value for number of cells or percentage (with statistics) is provided.

Methodology

Sample preparation

Mice were euthanized with CO_2 followed by perfusion with cold PBS. The indicated tissues were extracted and processed as specified:

Spleen and lymph nodes:
Lymph nodes were harvested from inguinal, mesenteric, cervical, and axillar areas. Spleen and lymph nodes were mashed and filtered through a 70-µm cell strainer. Red blood cells were removed using 5 ml of Erythrocyte lysis buffer (ammonium chloride 0.15 M, sodium bicarbonate 0.01 M, EDTA 0.0001 M) for 5 min. Cells were washed and counted.

Blood:

Blood was extracted from either the facial vein or the heart in living or euthanized mice, respectively. Red blood cells were removed using 5 ml of Erythrocyte lysis buffer for 5 min. Cells were washed and stained.

Colonic lamina propria:
T cells from colonic lamina propria were isolated as previously reported 67. Colon samples between the cecum and rectum were obtained and cleaned from fat and faeces. Tissues were cut longitudinally, washed with cold PBS and, then, cut transversally into 1cm-long fragments and mixed in pre-warmed 5 mM EDTA 14 mH Hepes 10% FBS PBS under shaking at 180 rpm for 30 min at 37ºC. After washing with PBS, tissue pieces were then minced and mixed in pre-warmed 25 mM Hepes 10% FBS RPMI supplemented with 300 U/ml collagenase type VIII (Sigma, C2139) under shaking at 180 rpm for 45 min at 37ºC. Digested tissue was filtered through a 70-μm cell strainer, washed with 5 mM EDTA 14 mH Hepes 10% FBS PBS and centrifuged at 650 x g for 5 min at room temperature (RT). To further enrich in leucocytes, supernatants were centrifuged in a 40%/70% Percoll gradient (Sigma, GE17-0891-01) at 750 x g for 20 min at RT without acceleration or brake. Isolated cells were washed with PBS and resuspended in 2% FBS RPMI for counting.
White adipose tissue:
Gonadal white adipose tissue was obtained from the mouse abdominal cavity and mixed in 2 mg/ml BSA 2% FBS RPMI supplemented with 2 mg/ml collagenase type II (Sigma, C6885) under shaking at 180 rpm for 40 min at 37ºC. Digested tissues were vertically rested to separate fat from the aqueous phases, which were obtained using a 18G syringe. Then, cell suspensions were filtered through a 70-μm cell strainer and washed with 2% FBS RPMI. Finally, erythrocytes were removed by incubation with a lysis buffer for 5 min at 4ºC, washed with 1 mM EDTA PBS and finally resuspended in 1 ml for counting.
Peyer's Patches:
Peyer's patches were harvested from the intestine and mashed into a 70-μm cell strainer. Cell suspension was centrifuged at 400 x g for 5 min at 4ºC. Finally, cell pellets were resuspended in 1 ml of 2% FBS RPMI for counting.

Liver:
Liver was harvested and cut into pre-warmed 25 mM Hepes 10% FBS RPMI supplemented with 0.4 mg/ml collagenase type VIII (Sigma, C2139) under shaking at 180 rpm for 45 min at 37ºC. Digested tissue was filtered through a 70-μm cell strainer and centrifuged at 350 x g for 5 min at 4 ºC. Red blood cells were removed using 5 ml of Erythrocyte lysis buffer for 5 min. Cells were washed and counted. To further enrich in leucocytes, supernatants were centrifuged in a 40%/70% Percoll gradient (Sigma, GE17-0891-01) at 1250 x g for 30 min at RT with acceleration on 6 and without brake. Isolated cells were washed with PBS and resuspended in ml 2% FBS RPMI for counting.

Bone marrow:
Femurs and tibias were collected. Cells from the bone marrow were obtained by centrifuging the bones at 6000 g for 1 minute. Red blood cells were removed using 5 ml of Erythrocyte lysis buffer (ammonium chloride 0.15 M, sodium bicarbonate 0.01 M, EDTA 0.0001 M) for 5 min. Cells were washed and counted.

To differentiate between live and dead, the cells were firstly stained with the Zombie NIR™ Fixable Viability Kit, the Zombie Yellow™ Fixable Viability Kit or the Ghost Dye™ Violet 540 for 20 min at 4ºC. Then, the cells were washed with FACS staining buffer (PBS supplemented with 2% fetal bovine serum and 1 mM EDTA) and incubated with Fc receptor blocker purified rat anti-mouse anti-CD16/CD32 (BD Biosciences, 553142) for 20 min at 4°C. Cells were then incubated with primary antibodies for 20 min at 4°C and were washed twice with FACS staining buffer. For intracellular staining, after staining for membrane markers, the cells were fixed and permeabilized using the FOXP3/Transcription Factor Staining Kit (eBioscience) for 20 min at RT and darkness.

Human samples:
Human blood samples were collected by venipuncture in an overnight fasting state. 3 ml of blood were collected in TransFix/ EDTA Vacuum Blood Collection Tubes (Cytomark, Buckingham, U.K.) and preserved until the day of staining and cell acquisition.
Cells were labelled by incubation with appropriate fluorescence-conjugated antibodies for 15 min at RT in the dark. Cells were then lysed with 2 mL of FACS lysing solution (BD Biosciences) for 10 minutes and centrifuged at 500 × g for 5 min at RT. Then, the cells were washed with 5 mL of PBS. The following antibodies were used for surface antigen staining.
Experiments with human samples were performed in a BD FACSymphony SORP flow cytometer (BD Biosciences). In order to generate comparable results among patients and over time, the photomultiplier voltages were adjusted to unlabeled lysed whole blood cells to obtain optimal photomultiplier tube voltages for the resolution of dim cell populations. The target values resulting of the optimization were used for subsequent calibrations to maintain instrument standardization. When possible, at least 40.000 events of CD4 population were acquired in order to reach the maximum Tregs events.

Instrument

Mouse samples were acquired in a 4-laser (Violet, blue, yellow-green, red) or a 5-laser Aurora analyzer (Cytek Biosciences).
Human samples were acquired in a BD FACSymphony SORP flow cytometer (BD Biosciences).

Software

Data were analyzed with the FlowJo v10.5.3 software (BD Biosciences).
Dimensional reduction and clustering analysis of flow cytometry data was done using OMIQ (Dotmatics).

Cell population abundance

For in vitro suppression and adoptive transfer experiments, Tregs KLRG1- and kTregs were sorted from the same mouse. Tregs were sorted from 5 young mice that had been previously injected with IL-2 + IL-33. Cells were gated as:
1. Lymphocytes in SSC-A / FSC-A plot
2. Alive cells as DAPI- in DAPI / FSC-A plot.
3. Single cells as a diagonal in FSC-H / FSC-A plot.
4. Tregs as CD4+CD25+ in CD25 / CD4 plot.
5. Tregs KLRG1- as KLRG1- in KLRG1 / FSC-A plot.
6. kTregs as KLRG1+ in KLRG1 / FSC-A plot.

For RNAseq, rTregs, aTregs and kTregs were sorted from the same mouse. Treg subsets were sorted from 4 young and 4 old mice. Cells were gated as:
1. Lymphocytes in SSC-A / FSC-A plot
2. Alive cells as DAPI- in DAPI / FSC-A plot.

3. Single cells as a diagonal in FSC-H / FSC-A plot.
4. Tregs as CD4+CD25+ in CD25 / CD4 plot.
5. rTreg as CD62L+ KLRG1- in KLRG1 / CD62L plot.
6. aTreg as CD62L- KLRG1- in KLRG1 / CD62L plot.
7. kTreg as CD62L- KLRG1+ in KLRG1 / CD62L plot.

After the sorting, the purity of sorted cells was checked in the same sorter. Purity was over 95% in all the sorted cells.

Gating strategy

The gating strategy for mouse sample was:
1. Lymphocytes in FSC-A / SSC-A plot.
2. Single cells as a diagonal line in FSC-H / FSC-A plot.
3. Alive cells in a FSC-A / Viability marker.
4. CD4+ cells in CD4 / CD8 plot.

Boundaries between positive and negative populations were stablished above 10^3 and when the two populations were clearly defined.

The gating strategy for human samples is illustrated in Extended Data Figure 6. Briefly:
1. Single cells as a diagonal line in FSC-H / FSC-A plot.
2. Lymphocytes in FSC-A / SSC-A plot.
3. Leukocytes (CD45+ cells) in SSC-A / CD45 plot. CD45+ cells were gated.
4. Lymphocytes in SSC-A / FSC-H plot. FCS-Hlo cells were gated.
5. Lymphocytes in SSC-A / CD45 plot. CD45hi cells were gated.
6. T cells in CD3 / CD19 plot. CD3+CD19- cells were gated.
7. CD4+ T cells in CD4 / CD8 plot.
8. CD25+ CD127- cells in CD25 / CD127 plot.
9. KLRG1+ cells in CD4 / KLRG1 plot.

☒ Tick this box to confirm that a figure exemplifying the gating strategy is provided in the Supplementary Information.
